# Tailored pc@zro₂@cellulose fiber composites as versatile materials for photocatalytic metal ion reduction and microbial deactivation

**DOI:** 10.1038/s41598-025-08629-4

**Published:** 2025-07-16

**Authors:** Reem. Mohammed, Shimaa M. Abdel-Moniem, Nabila S. Ammar, Mohamed A. El-Liethy, Hanan S. Ibrahim, Mohamed Eid M. Ali

**Affiliations:** 1 Physics Department, Faculty of Science, Aim Shams University, Cairo, Egypt; 2 Water Pollution Research Department, National Research Centre, El- Buhouth St., Dokki, Cairo, P.O. 12622, Egypt

**Keywords:** Photocatalysis, Fixed bed photocatalysts, ZrO_2_@cellulose fibers, Microbial deactivation, Heavy metal ions, Biophysical chemistry, Chemical modification, Environmental sciences, Environmental chemistry

## Abstract

Water scarcity and contamination pose significant challenges to public health globally. Addressing these issues requires innovative and sustainable water purification technologies. This study investigates novel fixed bed photocatalysts composed of ZrO_2_@cellulose fibers, combined with various photocatalytic materials (PCs), including ZnO, V_2_O_5_, Bi_2_S_3_, MoS_2_, and PANI, for the reduction of heavy metal ions and microbial deactivation in water. The composite materials were characterized using X-ray diffraction (XRD), Fourier-transform infrared spectroscopy (FTIR), scanning electron microscopy (SEM), N_2_ adsorption/desorption isotherms, and diffuse reflectance spectroscopy (DRS). The results revealed successful integration of the PCs with the ZrO_2_-cellulose matrix, facilitating efficient charge transfer and enhanced photocatalytic performance. The photocatalytic activity was evaluated through the reduction of Cr (VI) and Cu (II) ions in simulated wastewater under simulated sunlight irradiation. ZnO-4%@ZrO_2_@cellulose and Bi_2_S_3_-4%@ZrO_2_@cellulose demonstrated excellent performance, achieving high reduction rates of Cr (VI) and Cu (III) ions. Kinetic studies showed that the Cr (VI) and Cu (III) ions reduction followed first-order kinetics. The reduction rates were significantly higher for the ZnO, Bi₂S₃, MoS₂, and PANI composites compared to the ZrO₂-cellulose control. Proposed mechanisms elucidate the role of reactive oxygen species in damaging microbial structures. Overall, the results suggest that the developed fixed bed photocatalysts hold promise for efficient water purification.

## Introduction

The low-income and developed countries confront a problem related to the contamination of water resources with customary pollutants such as heavy metals or emerging micropollutants which are discharged due to human activity^1–3^. Water contamination by heavy metals poses a significant threat, especially in low-income and developing countries, where industrial activities and improper waste disposal lead to the accumulation of these toxic substances. Heavy metals, including lead, mercury, cadmium, arsenic, and chromium, are non-biodegradable and can persist in water bodies for extended periods. When ingested or absorbed by living organisms, they can accumulate in the body, causing severe health effects such as kidney damage, neurological disorders, developmental issues in children, and increased risk of cancer. In aquatic ecosystems, heavy metals disrupt biodiversity, harming aquatic life by affecting reproduction and growth^4–7^. Given the hazardous impact of heavy metals, there is an urgent need for effective removal techniques. Traditional methods like chemical precipitation, ion exchange, and filtration often struggle to achieve complete elimination and may produce secondary waste. Catalysis, in general, refers to the acceleration of a chemical reaction by a substance (catalyst) that is not consumed in the reaction. However, conventional catalysis often relies on thermal or chemical energy inputs, which may not be sustainable or selective enough for trace contaminant degradation.

Photocatalysis, on the other hand, is a specialized form of catalysis that utilizes light energy typically from sunlight or artificial UV sources to activate a photocatalyst, triggering redox reactions. This light-driven approach offers several distinct advantages over traditional catalysis. Photocatalysis enables non-selective and complete mineralization of various organic and inorganic pollutants, operates under ambient conditions, and eliminates the need for harsh chemical reagents or external energy sources, making it highly suitable for green chemistry and environmental remediation.

Recent advancements in nanotechnology and composite materials have enabled the development of multifunctional adsorbents and catalysts that can not only remove but also recover valuable metals from wastewater, contributing to circular economy goals. For example, Zhou et al. (2024) developed a hierarchical CoFe₂O₄@ZIF-67 hybrid for selective Cr(VI) removal and recovery under visible light, highlighting the potential of integrated catalytic-adsorptive systems for sustainable remediation efforts^8^.

 Wang et al., have demonstrated the superior capability of photocatalytic systems in achieving high degradation efficiencies for emerging micropollutants, further validating its potential as a sustainable alternative^9–11^. Utilizing light-activated catalysts, such as zinc oxide (ZnO) or titanium dioxide (TiO₂), photocatalysis drives redox reactions that can convert toxic heavy metals into less harmful forms or remove them entirely from water sources. By harnessing sunlight, this process offers a sustainable and energy-efficient solution for water purification, making it particularly suitable for low-income and resource-limited settings.

Zirconium dioxide (ZrO₂) has gained significant attention as a photocatalyst due to its unique combination of advantageous properties. One of its primary strengths is its exceptional thermal and chemical stability, allowing it to function effectively under demanding conditions for extended periods. Additionally, ZrO₂ exhibits remarkable chemical inertness, making it highly resistant to corrosion and degradation in harsh environments, such as in photocatalytic water treatment. ZrO₂ is also a non-toxic and environmentally friendly material, making it ideal for sustainable applications, particularly in pollutant degradation and water purification, without generating harmful by-products. Moreover, ZrO₂ can be synthesized into nanoparticles or nanostructures with large surface areas, providing more active sites for reactions and boosting its overall performance. Its versatility is further enhanced by its ability to form heterojunctions with other materials, which expand light absorption and synergistically improve catalytic activity. Due to these attributes, ZrO₂ is widely applied in photocatalytic processes, particularly in water treatment, where its stability, chemical resilience, and photocatalytic capabilities make it an effective solution for removing contaminants and pollutants^12,13^.

Despite its inherent advantages, ZrO₂ nanoparticles face certain limitations in photocatalytic performance, such as a narrow light absorption range, and relatively low quantum efficiency. These constraints hinder its effectiveness under visible light, limiting its broader application in solar-driven processes. To overcome these challenges and fully unlock the potential of ZrO₂, it is crucial to explore strategies that can enhance its photocatalytic activity. One promising approach is the formation of ZrO₂-based composites with other catalyst materials. By combining ZrO₂ with complementary photocatalysts, these composites can synergistically improve light absorption, electron transfer, and overall photocatalytic efficiency^12,13^.

Zinc oxide (ZnO) is a widely used n-type semiconductor characterized by a suitable band gap of 3.37 eV and high electron mobility, which facilitates rapid charge carrier transfer. When combined with zirconium dioxide (ZrO₂), ZnO enhances photocatalytic performance through effective electron transport^14–21^. Similarly, copper sulfide (CuS), a narrow band gap (1.2 eV) p-type semiconductor, extends light absorption into the visible spectrum and forms a p–n heterojunction with ZrO₂, promoting efficient charge separation. Further enhancement is achieved by incorporating molybdenum disulfide (MoS₂), whose layered structure and large surface area provide numerous active sites for photocatalytic reactions, while also supporting efficient charge transport^22^, Alongside this, Polyaniline (PANI), a conductive π-conjugated polymer, acts as both a photosensitizer and an electron mediator, enhancing charge mobility within the composite system^23^. Additionally, Bismuth sulfide (Bi₂S₃), with a narrow band gap of approximately 1.3 eV, also facilitate visible-light-driven activity^24^. Moreover, Vanadium pentoxide (V₂O₅) enhances redox reactions through its multivalent oxidation states and promotes oxygen vacancy formation, further improving photocatalytic efficiency^25^. Together, these enhancements include the creation of direct carrier conduction pathways, increased surface-to-volume ratios, improved charge transfer dynamics, and greater reactivity at the catalyst surface, often due to defect sites generated by nonstoichiometric oxygen. Such synergistic properties collectively promote higher rates of photocatalytic reactions and mineralization. Therefore, a comparative analysis will be performed to assess the influence of each photocatalyst on the ZrO₂-based system, in order to identify the most effective material for enhancing its photocatalytic activity. 

In addition to material selection, recent advances in photocatalytic technologies have introduced hybrid systems that integrate photocatalysts with microbial components. These systems leverage the synergistic interaction between photocatalysis and microbial degradation, improving the breakdown of organic and inorganic pollutants while enhancing sustainability through bioremediation mechanisms^26^.

Such hybrid systems combine the oxidative strength of photocatalysts with the metabolic capabilities of microorganisms, offering a dual-action pathway for pollutant removal. Microorganisms can degrade intermediates generated by photocatalysis or even assist in regenerating the photocatalyst surface by removing organic fouling. These bio-hybrid systems have shown enhanced performance in treating wastewater containing complex or recalcitrant compounds. For instance, as demonstrated by Kumar et al.^27^, microbial-assisted photocatalytic systems significantly improved the degradation efficiency of persistent pollutants while also contributing to process sustainability and cost-effectiveness.

Furthermore, progress in nanostructure design, heterojunction engineering, and defect modulation continues to expand the potential of photocatalysts for environmental remediation applications^28^.

Despite these advancements, the practical use of powdered photocatalysts is often limited by the difficulties associated with separating and recovering the particles from liquid phases. To address this challenge, integrating nanoscale building blocks into macroscopic structures via self-assembly has emerged as a highly effective strategy. This approach not only facilitates the immobilization or incorporation of nanoparticles (NPs) onto various substrates^29–31^ such as silica^32–34^, carbon nanomaterial^35,36^, metal-organic frameworks^37,38^, ceramics glass^39–41^, and polymers but also enhances the practical applicability of these photocatalysts. By leveraging self-assembly, the nanoparticles can be effectively stabilized and utilized in practical applications, bridging the gap between their advanced photocatalytic properties and real-world usability.

However, this immobilization process can lead to a notable reduction in photocatalytic activity, primarily due to the decrease in the effective surface area of the catalysts. Cellulose fibers present a promising alternative as a support material. Their natural biopolymer composition offers several advantages: they are biodegradable, non-toxic, biocompatible, readily available, and economically viable^42^. Utilizing cellulose fibers allows for the creation of a fixed bed of photocatalysts, which minimizes the risk of secondary contamination from metal nanoparticles and enhances recyclability, thereby reducing environmental impact. Consequently, cellulose fibers offer a robust substrate for developing antibacterial materials.

 Wang et al. developed PVA/kaolin/GO composite beads that exhibit notable adsorption-encapsulation synergy for metal removal^43^. However, these materials act as passive adsorbents and do not facilitate active degradation of contaminants. In contrast, the current study introduces a multifunctional photocatalytic platform that integrates visible-light-responsive ZrO₂-based nanocomposites with cellulose fiber immobilization. This system not only captures but also catalytically reduces or mineralizes heavy metals through redox reactions under ambient, solar-driven conditions. The synergistic combination of enhanced charge mobility, extended light absorption, and recyclable biopolymer support provides a significant advancement over conventional methods. It ensures long-term operational stability, reduces secondary pollution risk, and promotes true detoxification rather than mere containment of heavy metals.

In addition to photocatalytic and bio-hybrid approaches, other disinfection technologies such as ultrasonic treatment have been explored for microbial inactivation. Ultrasonic methods rely on acoustic cavitation, which generates localized high temperatures and pressures capable of damaging microbial cell walls, disrupting cellular components, and leading to microbial death. This technique is considered a green and efficient approach, especially when combined with other advanced oxidation processes. Recent studies have been demonstrated the potential of ultrasonic disinfection as a powerful tool for microbial control in wastewater treatment systems^27^. However, despite its advantages, ultrasonic treatment also has several limitations. These include high energy consumption, especially at large scale; limited penetration depth, making it less effective in turbid or high-solid-content waters; and relatively low microbial inactivation efficiency when used alone without synergistic processes. Additionally, the operational cost and maintenance of ultrasonic equipment can be a barrier for widespread implementation. In contrast, photocatalytic methods offer a more scalable and energy-efficient alternative, particularly under sunlight irradiation and when utilizing stable, reusable nanomaterials.

In contrast, our photocatalytic system harnesses solar energy to trigger redox reactions that detoxify metals, potentially converting toxic species like Cr (VI) to less harmful Cr (III), with minimal secondary wastes and improved environmental sustainability. Therefore, the proposed approach not only provides high removal efficiency but also aligns with the goals of decentralized, green, and low-cost water treatment solutions.

The primary aim of this work is to develop a novel photocatalytic system by preparing a ZrO_2_-based photocatalyst and forming nanocomposites with ZnO, CuS, Bi_2_S_3_, MoS_2_, V_2_O_5_, and PANI. This study further seeks to enhance the practical applicability of these photocatalysts by immobilizing them onto cellulose fiber polymers. The ultimate goal is to optimize the photocatalytic performance of these nanocomposites for the effective reduction of heavy metals in wastewater. By combining advanced photocatalyst materials with environmentally friendly and recyclable support, this research aims to address the limitations of traditional photocatalysts and provide a sustainable solution for wastewater treatment.

## Experimental

### Chemicals

Ammonium metavanadate (NH₄VO₃), cellulose acetate (C₇₆H₁₁₄O₄₉), and acetone (C_3_H_6_O) were purchased from Luba Chemicals and used without any pretreatment. Reagent-grade potassium dichromate (K₂Cr₂O₇, ≥ 99.0% purity) and copper (II) sulfate pentahydrate (CuSO₄·5 H₂O, ≥ 99.0% purity) were obtained from Sigma-Aldrich. Aniline (≥ 99% purity, Aldrich) was distilled under reduced pressure and stored at 10 °C prior to use. Ammonium persulfate ((NH₄)₂S₂O₈, APS), nitric acid (HNO₃), sulfuric acid (H₂SO₄), and cetyltrimethylammonium bromide (CTAB) were also supplied by Luba Chemicals and were of analytical grade. Distilled water was used throughout all preparation processes.

### Preparation methods

#### Preparation of V_2_O_5_ nanoparticles

V₂O₅ nanoparticles were synthesized using ammonium metavanadate as the precursor. In a typical procedure, 2 g of ammonium metavanadate (NH₄VO₃) was dissolved in 100 mL of distilled water under stirring. Subsequently, 1 mL of concentrated nitric acid (HNO₃) was added dropwise to the solution with continuous stirring, resulting in the formation of a red-colored suspension. The resulting precipitate was collected by filtration, thoroughly washed with distilled water to remove any impurities, and dried at 80 °C for 12 h. The dried powder was then calcined at 500 °C for 1 h in the air to obtain V₂O₅ nanoparticles.

#### Preparation of PANI nanoparticles

PANI was synthesized through a hydrothermal technique. In a standard procedure, 0.7288 g of CTAB was dissolved in 70 mL of water while stirring vigorously in an ice bath to create a homogenous solution. Next, 0.2 mL of freshly distilled aniline was added to this CTAB solution. Following this, 2.72 mL of 12 M HCl was introduced, and the mixture was stirred for 10 min. Subsequently, a solution containing 1.125 g of APS was gradually incorporated into the monomer solution while maintaining vigorous stirring. The entire mixture was then transferred into a Teflon vessel. The hydrothermal reaction was carried out at temperatures of 120 °C for 12 h, after which it was allowed to cool overnight. Finally, the product was thoroughly rinsed with deionized water and dried under a vacuum to eliminate any moisture content.

The other nanomaterials utilized in this study are zirconia (ZrO_2_) powder^44^; bismuth sulfide (Bi₂S₃)^15^, zinc oxide (ZnO)^19^, and molybdenum disulfide (MoS₂)^16^ were prepared in our previous work.

#### PC@ZrO_2_@Cellulose fibers

**Fig. 1:**
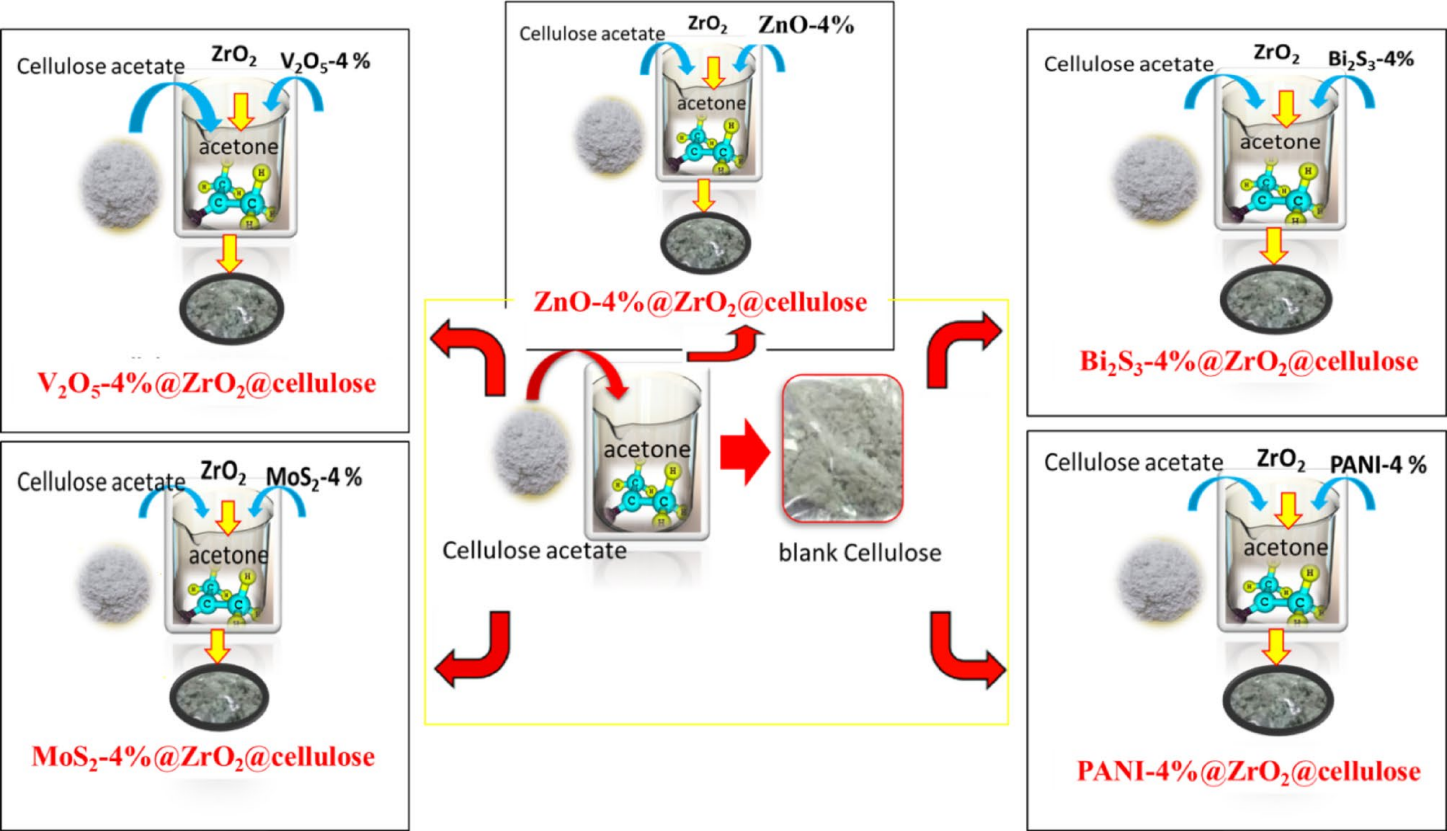
Scheme diagram for the preparation of fixed bed photocatalysts.

The experimental preparation method for PC@ZrO_2_@Cellulose fibers involved a systematic procedure as illustrated in Fig. 1. Initially, a solution comprising 1 gram of cellulose in 40 ml of acetone was prepared and stirred for 5 min to achieve uniform dispersion. Subsequently, 0.1 gram of ZrO_2_ nanoparticles were added to the cellulose-acetone solution and stirred for an additional 15 min to ensure proper integration. Following this, 0.04 g of the desired photocatalysts (ZnO, V_2_O_5_, Bi_2_S_3_, MoS_2_, or PANI, ) were individually incorporated into the solution, and stirring was continued for 30 min to facilitate homogeneous distribution. The solution was then combined with a mixture of 80 mL distilled water and 20 mL acetone to initiate the formation of cellulose fibers embedded with the photocatalyst-ZrO_2_ composite. Subsequent steps involved the preparation of cellulose fibers from the solution, thorough rinsing with water to remove residual solvent and unreacted materials, and drying until a constant weight was achieved. Finally, the dried cellulose fibers were weighed to determine their mass for further analysis and characterization. This method ensures the successful synthesis of PC@ZrO_2_@Cellulose fibers with embedded photocatalysts.

### Characterization techniques

The characterization of the fixed bed photocatalysts on support materials was conducted using several analytical techniques to elucidate their structural, morphological, and optical properties. Scanning Electron Microscopy (SEM) a Quanta 250 FEG (Field Emission Gun) model was employed to investigate the surface morphology and particle size distribution of the photocatalysts. Additionally, UV-Vis Diffuse Reflectance Spectroscopy (DRS) (a Jasco V-670 instrument from Japan) was utilized to analyze the optical properties and determine the bandgap energy of the photocatalysts. Fourier Transform Infrared Spectroscopy (FTIR) Jasco model (Japan) was employed to identify functional groups and chemical bonding present on the surface of the materials. Furthermore, X-ray Diffraction (XRD) (X’Pert PRO model) was employed to determine the crystal structure, phase purity, and crystallite size of the photocatalysts. A UV-Vis spectrophotometer model V730 (JASCO Japan) is employed to determine the concentration of the tested solution. Additionally, a thermo digital pH meter (USA) was utilized for measuring the solution pH.

### The photocatalytic activity

Initially, a solution containing 50 mL of simulated wastewater (Cr (IV) or Cu (II)) metal ions was introduced into a reactor containing a constant weight of the prepared photocatalytic material. The mixture was stirred for 30 min in the dark to allow for the adsorption of metal ions onto the surface of the photocatalyst and to establish the adsorption/desorption equilibrium. Subsequently, the reactor was placed under a solar simulator (Model: UVA CUBE 400, 400 W) to initiate the photocatalytic reaction for 2 h. The simulator provides a light spectrum closely matching the AM 1.5G standard. A UV cut-off filter was employed to eliminate wavelengths below 400 nm, allowing only visible and near-infrared light to reach the sample. The light intensity at the sample position was calibrated using a certified reference photodiode and was maintained at 100 mW/cm², corresponding to one sun intensity under standard testing conditions. Regular calibration was performed to ensure consistency and accuracy during the experiments.

At a certain time, interval, 3 mL of the reaction mixture was collected. The concentration of the simulated waste was measured in a UV/visible spectrophotometer by measuring the absorbance intensity at a wavelength of about 443 nm for hexavalent chromium (Cr (IV)). Meanwhile, the Copper concentration (Cu) in the filtrate was measured using inductively coupled plasma optical emission spectrometry (Agilent ICP-OES 5100, Australia), according to the previous method for the examination of water and wastewater^18^. The amount of pollutant adsorbed per unit mass of prepared materials (q_t_) at equilibrium is calculated as illustrated in Eq. 1:

1 $$\:{q}_{t}=\frac{\left({c}_{0}-{c}_{t}\right)v}{m}$$.

Where q _t_ (mg.g^− 1^) is the amount adsorbed per gram of adsorbent at time t (min), C_0_ is the initial concentration of simulated pollutant in the solution (mg.L^− 1^), C_t_ is the concentration at time (t) (mg.L^− 1^), m is the total addition amount of the adsorbent sample (g), and V is the volume of simulated waste solution (L). The percentage of the photodegraded molecules is calculated using the following equation (Eq. 2).

2 $$\:photoreduction\:\%,\:Degradation\:\text{\%}=\frac{{c}_{0}-{c}_{t}}{{c}_{0}}\times\:100$$

*C*_*o*_ and *C*_*t*_ are the initial concentration and concentration of simulated waste at time *t*, respectively.

### The photocatalytic microbial deactivation

The photocatalytic microbial deactivation of a fixed bed photocatalyst (PC) was evaluated against different microorganisms at various time intervals. The microorganisms tested included *Escherichia coli* (ATCC 35150), *Salmonella Typhimurium* (ATCC 14028), and *Bacillus subtilis* (ATCC 4342). Stock cultures of these microorganisms, stored at -20 °C, were revived by inoculating them into Tryptic Soy Broth (TSB) (Merck, Germany) and incubating for 18–24 h at 37 °C. The microbial suspensions were then diluted in 10 mL of sterile distilled water, resulting in initial microbial concentrations of approximately 10⁵–10⁶ CFU/mL. *E. coli* was determined on Rapid HiColiform agar. The typical *E. coli* colonies appeared in blue-green. While *Salmonella* spp. was enumerated on HiCrome improved salmonella agar plates. Positive *Salmonella* colonies appeared in pink to red color and light pink. However, *Bacillus subtilis* was determined on Hicrome Bacillus agar supplemented with polymyxin. The typical colony of *Bacillus subtilis* appeared in yellowish green to green color. All cultural plates were incubated at 37^o^C for 24–48 h. All the used media were purchased from HiMedia, India.

## Results and discussion

### Characterization of fixed beds photocatalysts

**Fig. 2:**
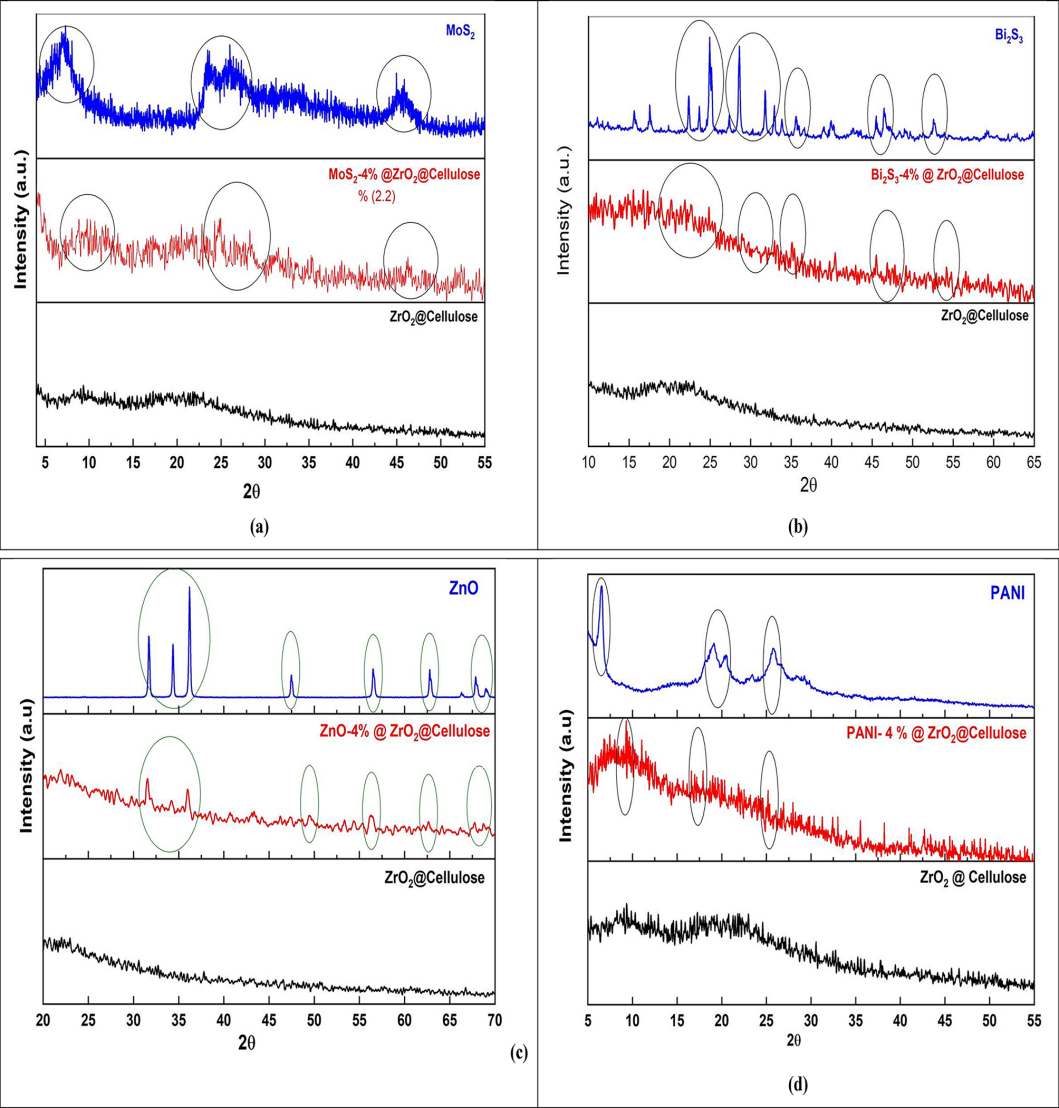
(**a**-**e**): The X-ray diffractometer (XRD) patterns of photocatalytic fixed bed for (**a**) MoS_2_-4%@ZrO_2_@Cellulose (**b**) Bi_2_S_3_-4%@ZrO_2_@Cellulose (**c**) ZnO 4%@ZrO_2_@Cellulose (**d**) PANI-4%@ZrO_2_@Cellulose, (**e**) V_2_O_5_-4%@ZrO_2_@Cellulose fixed beds.

The XRD patterns for fixed bed photocatalysts of ZrO_2_@Cellulose, ZnO-4%@ZrO_2_@Cellulose, V_2_O_5_-4%@ZrO_2_@Cellulose, Bi_2_S_3_-4%@ZrO_2_@Cellulose, MoS_2_-4%@ZrO_2_@Cellulose, and PANI-4%@ZrO_2_@Cellulose are depicted in Fig. 2.

As shown, the XRD pattern of the ZrO_2_@cellulose fiber revealed a diffraction pattern consistent with an amorphous structure. The absence of discernible peaks suggests that the ZrO_2_ nanoparticles embedded within the cellulose matrix do not exhibit long-range order, indicative of an amorphous phase. This observation aligns with previous reports highlighting the amorphous nature of ZrO_2_@cellulose composites^45^. For the MoS_2_ XRD spectrum (Fig. 2a), distinct diffraction peaks corresponding to MoS_2_ nanosheets were observed at 2θ angles of 7.5°, 33.3°, 39°, 49.8°, and 58°. These peaks are characteristic of the (002), (100), (103), (105), and (110) crystal planes of MoS_2_, respectively^16,18^. The presence of these peaks confirms the crystalline nature of MoS_2_ nanosheets. Upon analyzing the XRD pattern of the MoS_2_-4%@ZrO_2_@cellulose fixed bed structure, it was observed that the characteristic diffraction peaks of MoS_2_ were retained. However, minor alterations in peak intensities were noted, indicating interactions between MoS_2_ nanosheets and the cellulose fiber matrix. This phenomenon suggests potential changes in the crystalline arrangement or interfacial effects induced by the incorporation of MoS_2_ nanoparticles within the cellulose matrix. Such interactions could lead to modifications in the structural properties and surface characteristics of the composite, which may have implications for its photocatalytic performance.

XRD pattern of Bi_2_S_3_ NSs and Bi_2_S_3_-4%@ZrO_2_@cellulose fiber is shown in Fig. 2b. As discussed previously, the Bi_2_S_3_ NSs exhibits diffraction peaks at 2θ values of 15.6, 17.5, 22.1, 23.6, 25, 27.3, 28.5, 31.86, 32.9, 33.96, 35.69, 36.61, 39.13, 40.06, 42.69, 45.60, 46.60, 48.38, 49.16, 51.41, 52.71, 52.92, 54.67 and 59.29 correspond to (020), (120), (220), (101), (111), (021), (230), (221), (301), (311), (240), (231), (041), (141), (421), (002), (431), (060), (251), (222), (312) (061), (232) and (640) diffraction planes, respectively^15^. Analysis of the XRD pattern of the Bi_2_S_3_-4%@ZrO_2_@cellulose fixed bed structure revealed noticeable changes in the characteristic diffraction peaks compared to pristine Bi_2_S_3_. The diffraction peaks associated with Bi_2_S_3_ exhibited decreased intensity and broadening, indicative of a transition toward an amorphous phase. This transformation is attributed to the presence of ZrO_2_@cellulose, suggesting interactions between Bi_2_S_3_ NSs and the composite matrix. The amorphization of Bi_2_S_3_ within the composite structure may result from the incorporation of ZrO_2_ nanoparticles, which could disrupt the crystalline arrangement and induce disorder in the material.

Figure 2c showed characteristic peaks for ZnO in ZnO-4%@ZrO_2_@cellulose fiber composite.

The XRD pattern of the ZnO NRs revealed distinct diffraction peaks at 2θ values corresponding to the hexagonal wurtzite structure of ZnO. The observed peaks at 31.7°, 34.42°, 36.24°, 47.5°, 56.554°, 62.83°, 67.9°, and 69.01° can be indexed to the (100), (002), (101), (102), (110), (200), (201), and (004) crystal planes, respectively. These findings are consistent with the card number (JCPDS Card No. 96-900-4181) for ZnO with a wurtzite hexagonal phase^20^. Upon analyzing the XRD pattern of the ZnO-4%@ZrO_2_@cellulose fixed bed, it was observed that the characteristic diffraction peaks of ZnO NRs were preserved. However, slight reductions in peak intensities were noted, indicating potential interactions between ZnO NRs and the cellulose fiber matrix.

Figure 2d illustrates the XRD pattern of the PANI nanoparticles which exhibited major characteristic peaks at 2θ values of approximately 19°, 20.5°, 20.2°, and 25.7°. These peaks correspond to the diffraction from the crystalline regions of the PANI structure. Additionally, a broad, intense peak at 2θ = 6.5° was observed, which is attributed to the presence of long PANI chains and a more ordered structure within the composite^46^. This peak indicates the existence of extended π-conjugation along the polymer chains, suggesting a degree of structural regularity and alignment. The XRD pattern for the PANI-4%@ZrO_2_@cellulose fixed bed structure revealed the retention of major characteristic peaks corresponding to PANI nanoparticles. The positions of these peaks are consistent with those observed for PANI alone, indicating the preservation of the crystalline structure within the fixed bed structure.

The XRD pattern of the V_2_O_5_ nanoparticles exhibited three prominent diffraction peaks at 2θ values of approximately 8°, 25.6°, and 34.5°, corresponding to the (200), (110), and (101) diffraction planes, respectively (F.g.2e). These peaks are consistent with the orthorhombic crystal structure of V_2_O_5_, as confirmed by comparison with the standard JCPDS data (card no: 01–070-8747)^47^. The absence of impurity peaks in the XRD pattern indicates the formation of crystalline V_2_O_5_ samples. The XRD pattern of the V_2_O_5_-4%@ZrO_2_@cellulose fixed bed structure revealed that the characteristic diffraction peaks of V_2_O_5_ nanoparticles were retained confirming its introduction in fixed bed PC.

**Fig. 3:**
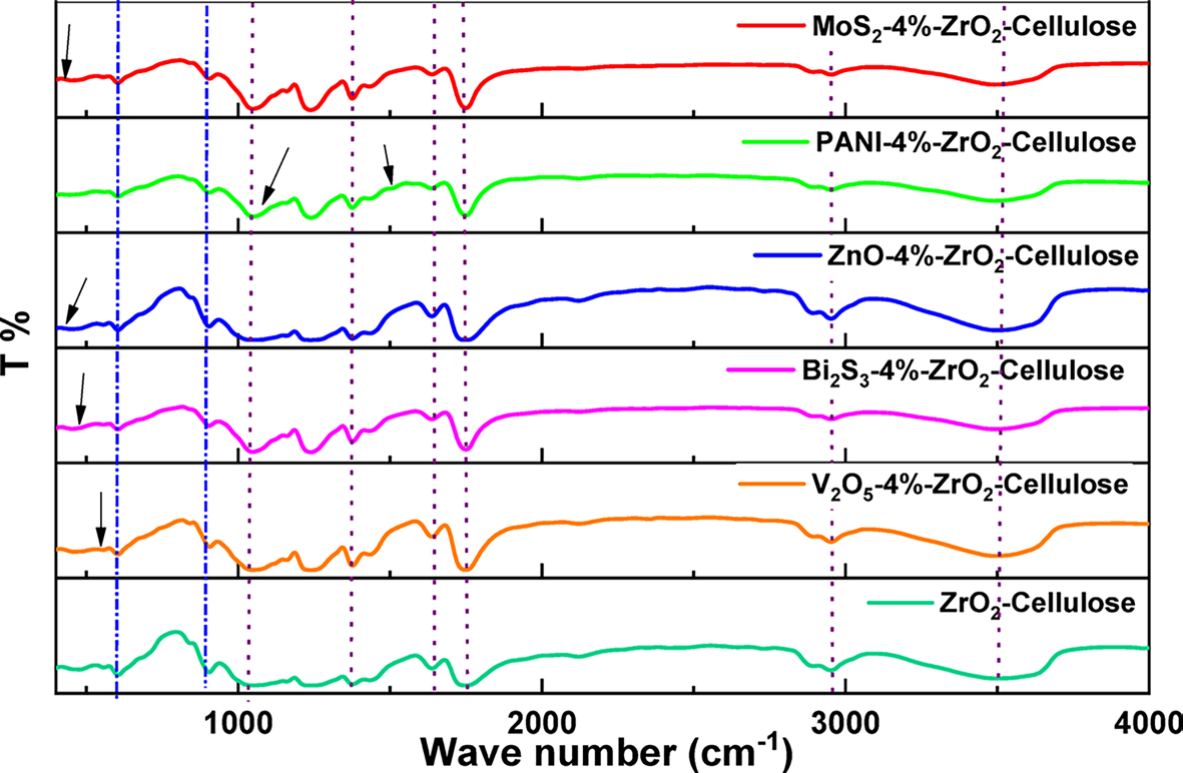
FTIR spectra of photocatalytic fixed bed for ZrO_2_@Cellulose, ZnO-4%@ZrO_2_@Cellulose, V_2_O_5_-4%@ZrO_2_@Cellulose, Bi_2_S_3_-4%@ZrO_2_@Cellulose, MoS_2_-4%@ZrO_2_@Cellulose, and PANI-4%@ZrO_2_@Cellulose.

Figure 3 showed FTIR spectra of photocatalytic fixed bed fibers of ZnO-4%@ZrO_2_@Cellulose, V_2_O_5_-4%@ZrO_2_@Cellulose, Bi_2_S_3_-4%@ZrO_2_@Cellulose, MoS_2_-4%@ZrO_2_@Cellulose, PANI-4%@ZrO_2_@Cellulose fiber. The FTIR analysis of the ZrO_2_@cellulose fiber composite unveiled distinct transmission peaks indicative of its intricate chemical composition and structural characteristics. Specifically, transmission peaks were observed at 3556 cm^− 1^, associated with O-H stretching vibrations typical of hydroxyl groups present in both cellulose and ZrO_2_^48^. The peak at 2952 cm^− 1^ corresponds to the asymmetric stretching vibration of C-H bonds, likely originating from the cellulose component. At 1745 cm^− 1^, the peak suggests the presence of carbonyl groups (C = O stretching), potentially from ester linkages within cellulose. Additionally, the peak at 1637 cm^− 1^ may indicate the bending vibration of O-H groups from residual moisture^49^. The presence of ether linkages in cellulose is implied by the peak at 1037 cm^− 1^ (C-O stretching). The band at 1410 cm^− 1^ is attributed to the C–C stretching^50^. Peaks at 613 cm^− 1^ and 901 cm^− 1^ are attributed to bending and stretching vibrations of Zr-O bonds in ZrO_2_ nanoparticles, confirming their integration within the cellulose matrix^51^. These comprehensive findings underscore the complex interplay between cellulose and ZrO_2_.

Furthermore, additional peaks emerged at 463 cm⁻¹, 554 cm⁻¹, 443 cm⁻¹, and 506 cm⁻¹, corresponding to the presence of MoS_2_, Bi_2_S_3_, ZnO, and V_2_O_5_, respectively. These peaks highlight the successful incorporation of these photocatalysts into the cellulose-ZrO_2_ matrix.

In addition, the FTIR spectrum displayed characteristic peaks for PANI at 1079 cm⁻¹ (nitrate group), 2346 cm⁻¹ (C-O bond), 1600 cm⁻¹ (C = C stretching), and 1524 cm⁻¹ (N-H bond). The presence of these peaks indicates that PANI retains its functional groups even after being incorporated into the composite.

However, no additional peaks corresponding to the introduced PCs were detected in the FTIR spectra. This suggests that the incorporation of ZnO, V_2_O_5_, Bi_2_S_3_, MoS_2_, and PANI did not introduce new functional groups or alter the major chemical composition of the composite material. Instead, the observed shifts in existing peaks may imply changes in the bonding interactions within the composite structure induced by the presence of these photocatalysts.

**Fig. 4:**
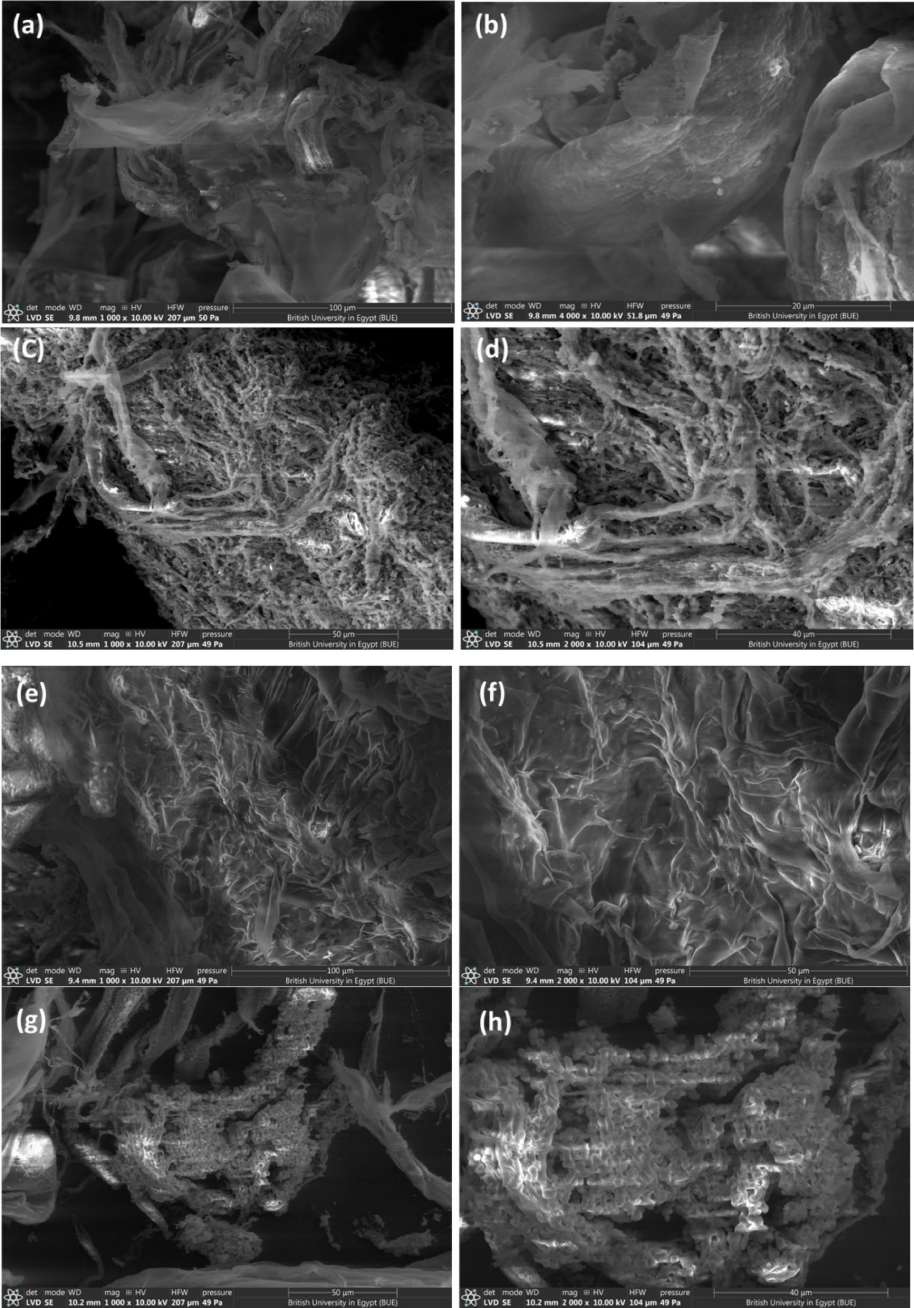

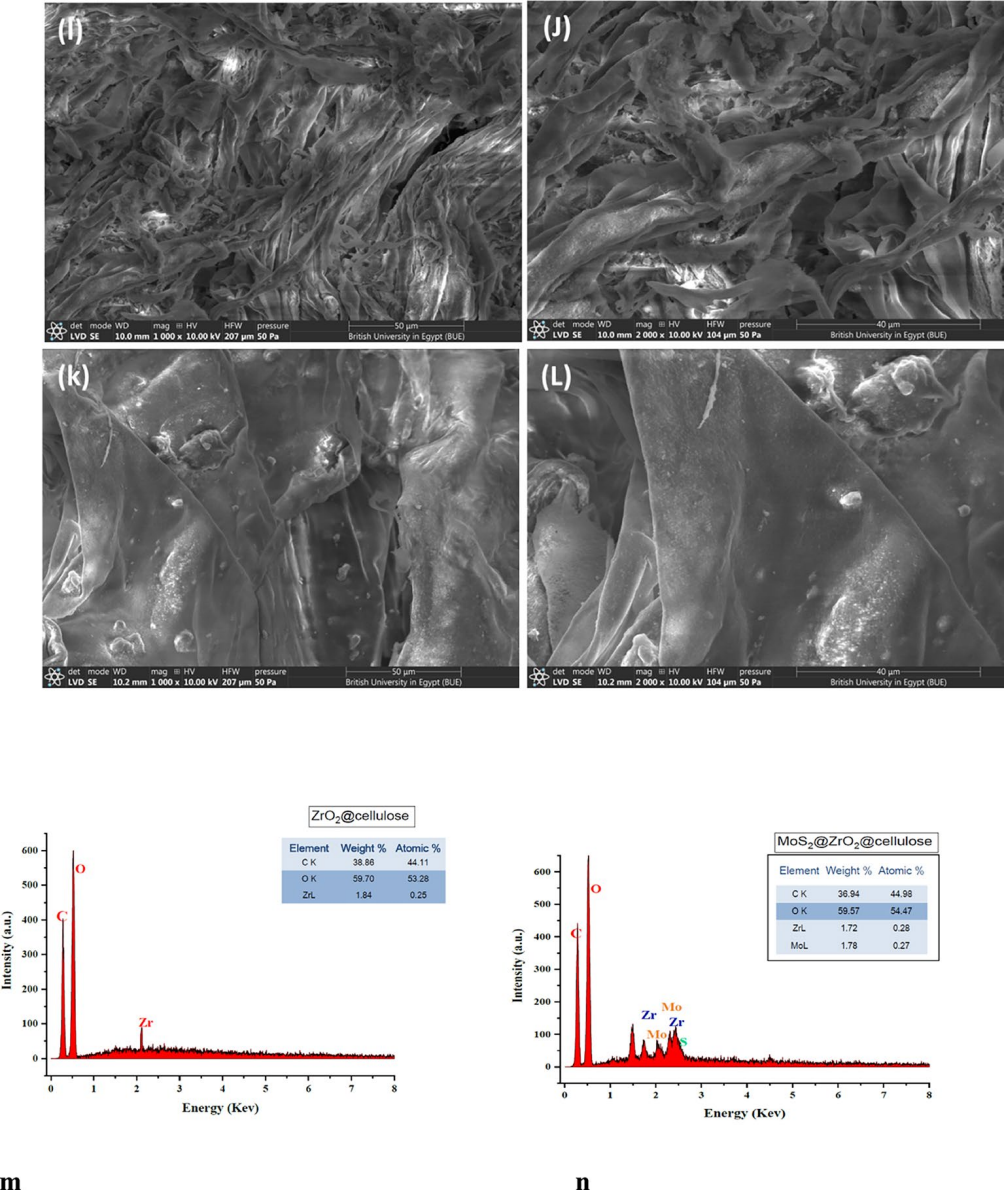

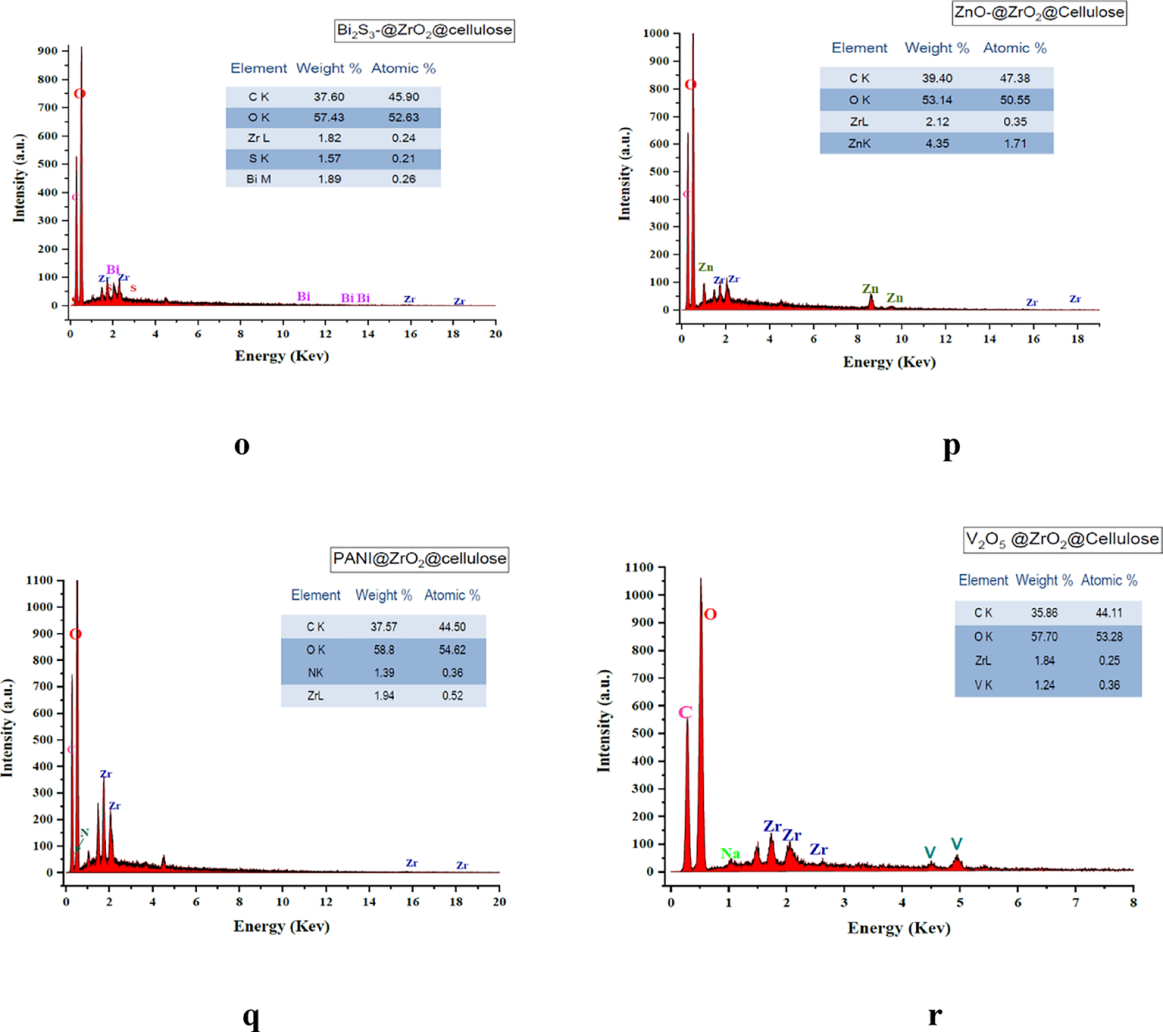
The SEM images of (**a**,** b**) ZrO_2_@Cellulose, (**c**,** d**) ZnO-4%@ZrO_2_@Cellulose, (**e**,** f**) V_2_O_5_-4%@ZrO_2_@Cellulose, (**g**,** h**) Bi_2_S_3_-4%@ZrO_2_@Cellulose, (**I**,** J**) MoS_2_-4%@ZrO_2_@Cellulose, (**k**,** l**) PANI-4%@ZrO_2_@Cellulose, EDX analysis of of (**m**) ZrO_2_@Cellulose, (**n**) ZnO-4%@ZrO_2_@Cellulose, (**o**) V_2_O_5_-4%@ZrO_2_@Cellulose, (**p**) Bi_2_S_3_-4%@ZrO_2_@Cellulose, (**q**) MoS_2_-4%@ZrO_2_@Cellulose, (r) PANI-4%@ZrO_2_@Cellulose.

Moreover, SEM images of the different fixed bed photocatalysts (PCs) were obtained and presented in Fig. 4. These images illustrate the close interaction between ZrO_2_ nanoparticles and the cellulose matrix. The SEM images revealed smoothly aggregated sheets of cellulose fibers for the ZrO_2_@cellulose fiber (Fig. 4a-b, which serves as the support matrix for the PCs facilitating efficient catalytic activity.

In Fig. 4c-d, SEM images depict the fixed bed structure of the ZnO-4%@ZrO_2_@cellulose PC. The ZnO NRs were observed to be densely loaded on the surface of ZrO_2_@cellulose fibers. This observation indicates successful immobilization and distribution of ZnO NRs onto the cellulose support matrix, facilitated by the presence of ZrO_2_ nanoparticles. The contact between ZnO NRs and the ZrO_2_@cellulose fibers enhances the accessibility of active sites and promotes synergistic interactions between the photocatalytic components.

In Fig. 4e-f, the SEM images depict the fixed bed structure of the V_2_O_5_-4%@ZrO_2_@cellulose PC. Notably, rough sheets of the fixed bed are observed, indicating the presence of well-dispersed V_2_O_5_ nanoparticles on the surface of cellulose fibers. The rough morphology suggests a high surface area and effective exposure of V_2_O_5_ particles, which is favorable for catalytic activity.

In Fig. 4g-h, the SEM images illustrate the fixed bed structure of the ZrO_2_@cellulose fiber loaded with Bi_2_S_3_ nanosheets. Notably, ZrO_2_@cellulose fibers are observed to be uniformly covered with Bi_2_S_3_ nanosheets. This observation indicates successful dispersion and distribution of Bi_2_S_3_ within the cellulose matrix, facilitated by the presence of ZrO_2_ nanoparticles. The homogeneous distribution of Bi_2_S_3_ nanosheets on the surface of ZrO_2_@cellulose fibers suggests strong interactions between the components and effective immobilization of the PC.

**Fig. 5:**
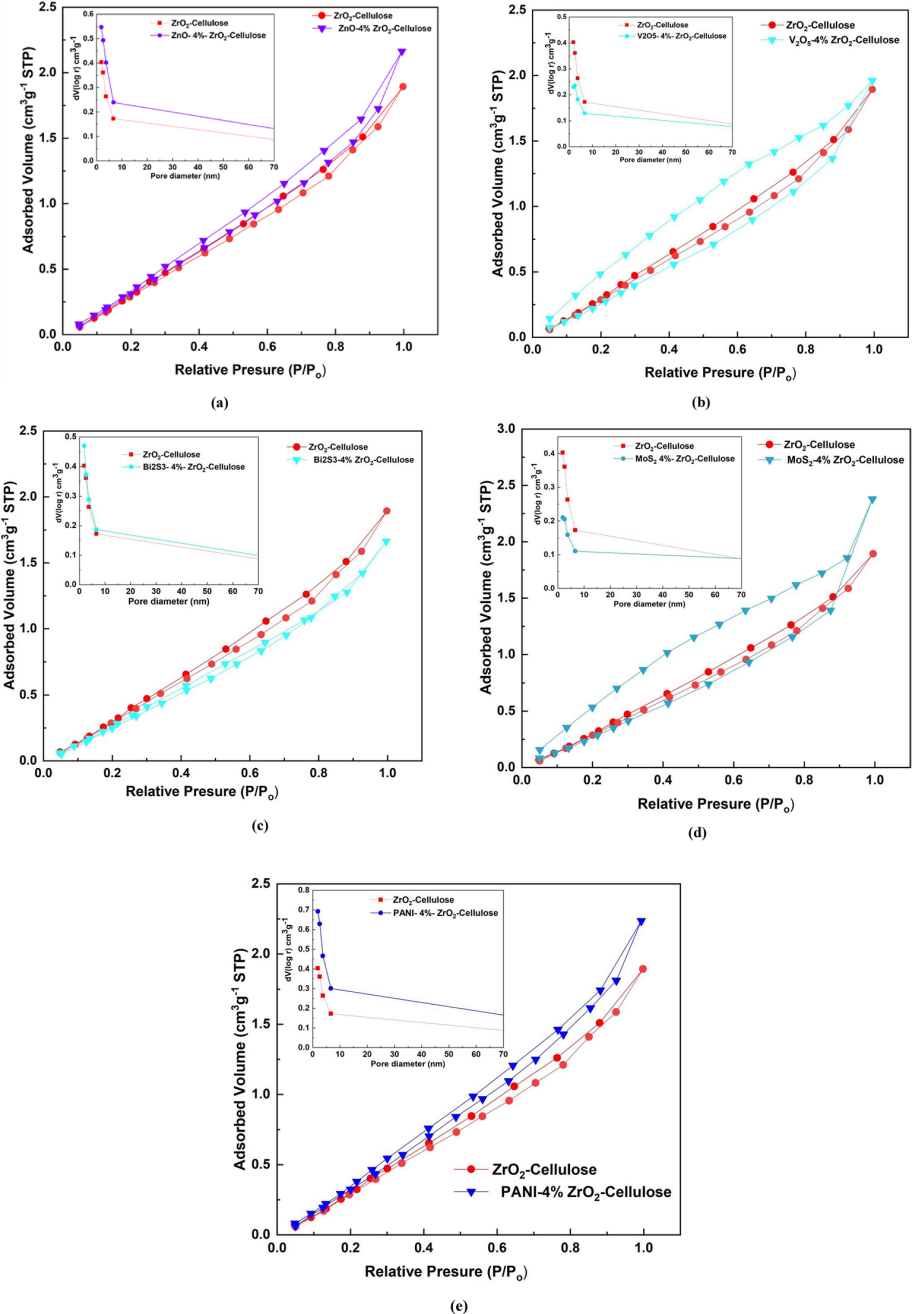
(**a**-**e**) The N_2_ adsorption-desorption isotherm for (**a**) ZnO − 4%@ZrO_2_@Cellulose fiber (**b**) V_2_O_5_-4%@ZrO_2_@Cellulose fixed bed fiber (**c**) Bi_2_S_3_-4%@ZrO_2_@Cellulose fixed bed fiber (**d**) MoS_2_-4%@ZrO_2_@Cellulose fixed bed fiber (e) PANI-4%@ZrO_2_@Cellulose fixed bed fiber.

In Fig. 4i-j, the SEM images depict the fixed bed structure of the MoS_2_-4%@ZrO_2_@cellulose PC. Notably, homogeneous sheets of ZrO_2_@cellulose are observed, indicating the well-distributed presence of MoS_2_ nanosheets. This observation is consistent with the earlier observation of Bi_2_S_3_ distribution within the fiber matrix. In Fig. 4k-l, the SEM images illustrate the fixed bed structure of the ZrO_2_-cellulose fibers loaded with PANI particles. Notably, dispersed PANI particles are observed on the cellulose fibers. Overall, the interaction between the PCs and the ZrO_2_@cellulose fibers promotes efficient charge transfer, enhances the accessibility of active sites, and improves photocatalytic performance. The fundamental conformation of the different fixed bed photocatalysts (PCs) was investigated using EDX analysis and displayed in Fig. 5m-r. The data obtained shows detection of carbon and oxygen and Zr in all samples with relative atomic and weight ratio; Carbon (C) (35–38%), Oxygen (O) (52–56%) and Zr (1.84–2.12%), representing the dominant elements that existed in fixed bed ZrO_2_/cellulose. Additional to the dominant elements, significant amounts of Mo and S are displayed in Fig. 5n indicating integration of MoS_2_ in ZrO_2_/cellulose fixed bed, meanwhile, extra materials of Bi and S are presented in Bi_2_S_3_-4%@ZrO_2_@cellulose fiber (See Fig. 5O) and confirmed integration of Bi_2_S_3_ in fixed bed. Moreover, Zn, N and V are detected in ZnO-4%@ZrO_2_@cellulose fiber, PANI-4%@ZrO_2_@cellulose fiber and V_2_O_5_-4%@ZrO_2_@cellulose fiber as shown in Fig. 5p, q and r, respectively. The above-mentioned data confirm the efficacious incorporation of PCs forming different fixed bed photocatalyst of ZnO-4%@ZrO_2_@Cellulose, V_2_O_5_-4%@ZrO_2_@Cellulose, Bi_2_S_3_-4%@ZrO_2_@Cellulose, MoS_2_-4%@ZrO_2_@Cellulose, and PANI-4%@ZrO_2_@Cellulose.

Figure 5 presented the N_2_-adsorption/desorption isotherms for the various photocatalytic fixed bed fibers, including ZnO-4%@ZrO_2_@cellulose, V_2_O_5_-4%@ZrO_2_@cellulose, Bi_2_S_3_-4%@ZrO_2_@cellulose, MoS_2_-4%@ZrO_2_@cellulose, and PANI-4%@ZrO_2_@cellulose. These isotherms provide crucial insights into the surface area, pore diameter, and volume of the composite materials, which are vital parameters influencing their photocatalytic performance. The observed IV-type isotherm with an H_3_ hysteresis loop confirms the mesoporosity of the prepared fixed-bed photocatalysts. The calculated BET-surface area, pore size, and pore volume are summarized in Table 1.

The results show significant changes in surface properties, which are indicative of the interaction between the ZrO_2_@Cellulose matrix and each photocatalyst. The pristine ZrO_2_@Cellulose composite has a surface area of 333 m².g^− 1^, a total pore volume of 0.255 cm³ g^− 1^, and a mean pore diameter of 1.72 nm. This reflects the porous structure of the composite, with ZrO_2_ nanoparticles well dispersed within the cellulose fibers. The observed pore structure is expected to facilitate catalytic and adsorption processes.

Upon incorporating 4% Bi_2_S_3_, the surface area increases slightly to 337 m² g^− 1^, while the total pore volume rises to 0.289 cm³ g^− 1^, and the pore diameter increases to 1.96 nm. These changes suggest that Bi_2_S_3_ enhances the porous structure of the ZrO_2_@Cellulose matrix, likely due to a favorable interaction that preserves the surface area while slightly enlarging the pores. The minimal change in pore size and surface area suggests that the composite retains its porous nature, making Bi_2_S_3_ a potentially effective photocatalyst for this system.

The incorporation of 4% ZnO results in a substantial increase in surface area to 405 m².g^− 1^, with a total pore volume of 0.373 cm³ g^− 1^ and a pore diameter of 2.07 nm. The increase in surface area suggests that ZnO is well-dispersed within the matrix, enhancing the surface accessibility. This improved porosity is likely due to the introduction of ZnO NRs, which may prevent agglomeration and maintain an open structure, making the composite highly suitable for catalytic applications requiring large surface areas^19^. The addition of 4% PANI leads to the most significant increase in surface area, reaching 543 m² g^− 1^, along with a total pore volume of 0.459 cm³ g^− 1^ and a pore diameter of 1.9 nm. The high surface area is likely due to the polymeric nature of PANI, which introduces additional active sites and promotes the formation of a highly porous network^49^. This enhancement in both surface area and pore volume makes the PANI-incorporated composite highly attractive for adsorption and photocatalytic applications, where a large surface area is essential.

On the other hand, with the addition of 4% V_2_O_5_ into the ZrO_2_@Cellulose matrix, the surface area drops to190 m² g^− 1^, and the total pore volume decreases to 0.196 cm³ g^− 1^, while the pore diameter increases to 2.93 nm. This indicates that V_2_O_5_ leads to partial blockage of surface sites and a reduction in porosity, with the increase in pore diameter suggesting the formation of larger pores, potentially due to V_2_O_5_-induced agglomeration of ZrO_2_ particles. Similarly, the addition of 4% MoS_2_ results in a surface area reduction to 180 m² g^− 1^ and a total pore volume of 0.204 cm³ g^− 1^, with a substantial increase in pore diameter to 3.53 nm. Like V_2_O_5_, MoS_2_ appears to block surface sites and reduce overall porosity, likely due to its layered structure and tendency to agglomerate. However, the larger pore size in the MoS_2_-incorporated composite may facilitate the diffusion of larger molecules, making it suitable for applications where diffusion control is critical, despite the reduced surface area.

Overall, the results show that the differences in surface area and pore diameter reflect the unique interactions between each photocatalyst and the ZrO_2_@Cellulose matrix, influencing the potential applicability of these composites in various photocatalytic and adsorption processes.

**Table 1:** Surface area, textural properties, and band gaps for different fixed bed PCs.

Sample ID	Surface area (m^2^/g)	Total pore volume (cm^3^g^− 1^)	Mean pore diameter (nm)	E_g_ (eV)
ZrO_2_@Cellulose	333	0.255	1.72	4.4–2.8
V_2_O_5_-4%@ZrO_2_@Cellulose	190	0.196	2.93	4.35–1.7
Bi_2_S_3_-4%@ZrO_2_@Cellulose	337	0.289	1.96	3.9–1.5
ZnO-4%@ZrO_2_@Cellulose	405	0.373	2.07	4.5 − 2.82
PANI-4%@ZrO_2_@Cellulose	543	0.459	1.9	4.17–1.6
MoS_2_-4%@ZrO_2_@Cellulose	180	0.204	3.53	4.2–3.46

**Fig. 6:**
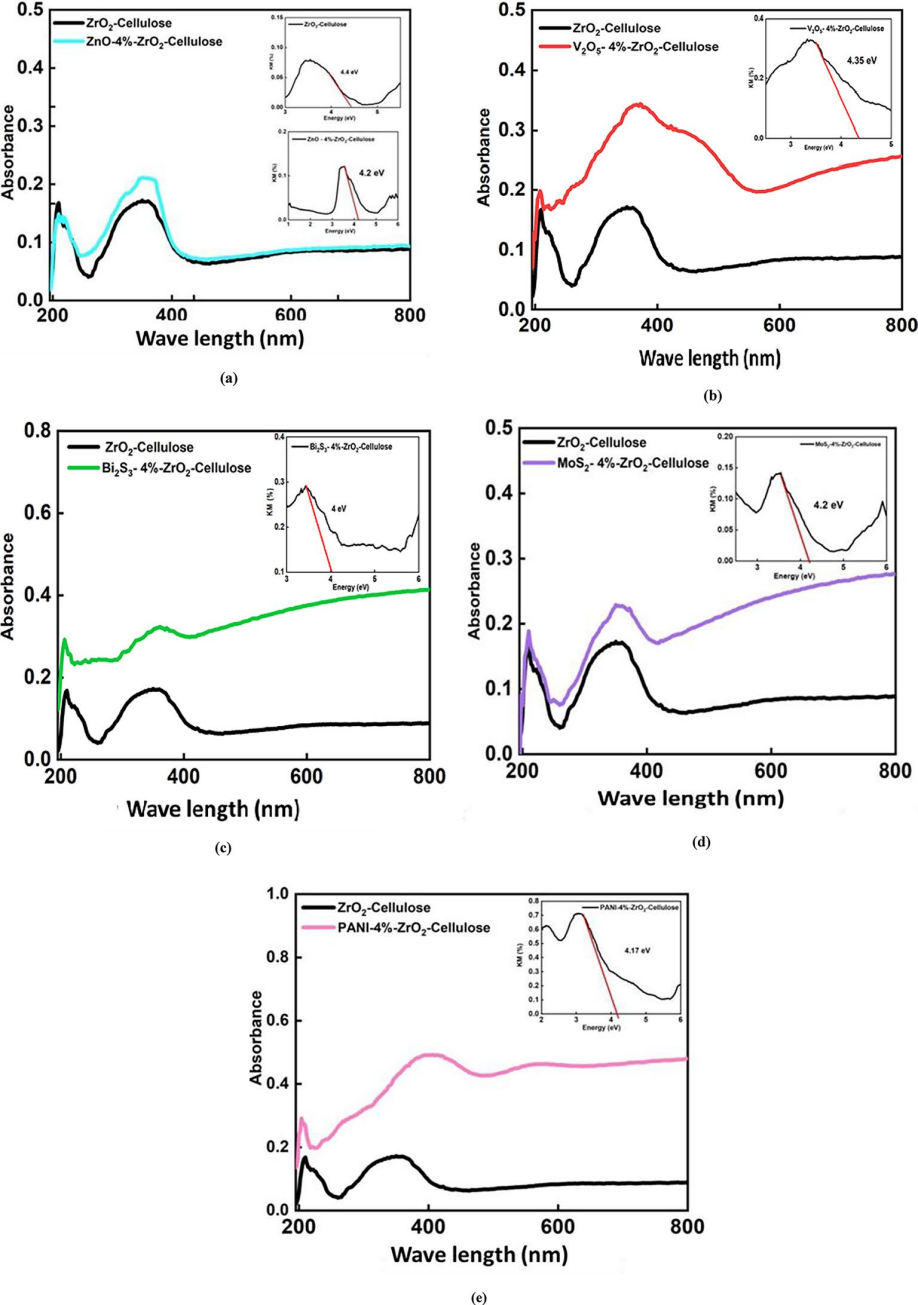
Optical spectra for (**a**) ZnO-4%@ZrO_2_@Cellulose, (**b**) V_2_O_5_-4%@ZrO_2_@Cellulose, (**c**) Bi_2_S_3_-4%@ZrO_2_@Cellulose, (**d**) MoS_2_-4%@ZrO_2_@Cellulose, and (**e**) PANI-4%@ZrO_2_@Cellulose fixed bed fibers.

The Diffuse Reflectance Spectroscopy (DRS) analysis provides valuable insights into the optical properties and bandgap energies (E _g_) of the prepared fixed bed composites. As illustrated in Fig. 6, the DRS spectra for each fixed bed PC exhibit two prominent absorption peaks, indicative of the materials’ optical bandgap (E _g_). The first absorption peak corresponds to the onset of electronic transitions from the valence band to the conduction band, representing the direct bandgap energy. The second peak may be attributed to indirect transitions or higher energy electronic states within the materials, and it could be associated with defect states within the nanocomposite structure. Defects, such as vacancies, can introduce localized electronic states with energy levels that manifest as absorbance peaks in the UV spectrum^49,52^.

The band gap values were determined using the Kubelka-Munk function (Eq. 3, Eq. 4) By plotting (αhν) ² against hν and identifying the tangent, the band gap energy (E_g_) was determined, as illustrated in Fig. 6b, c.

3 $$\:F\left(R\right)=\frac{{\left(1-R\right)}^{2}}{2R}=\frac{k}{5}\cong\:\alpha\:$$

4 $$\:\alpha\:\cong\:c\frac{{\left(h\nu\:-{E}_{g}\right)}^{n}}{h\nu\:}$$

Where (R) represents the Kubelka-Munk function, is the reflectance of the material, hν is the photon energy, h is the Planck’s constant (4.136 × 10^−15^ eV s^−1^), C is a fitting constant of the model, E_g_ is the band gap energy (eV), and n is the constant that depends on the type of optical transition (direct or indirect), where *n* = 2 for direct transitions and *n* = 1/2 for indirect transitions.

The ZrO_2_@cellulose composite itself showed a direct band gap ranging from 4.4 eV to 2.8 eV, which is typical for such ceramic and polymer composites.

Upon the introduction of 4% V_2_O_5_ to the ZrO_2_@cellulose composite, a significant reduction in the band gap was observed. The direct band gap was measured at 4.35 eV, while the indirect band gap decreased to 1.7 eV. The narrowing of the band gap suggests that V_2_O_5_ facilitates charge transfer within the composite, lowering the energy required for electronic transitions. This indicates that the inclusion of V_2_O_5_ not only alters the electronic structure of the composite but also creates new states within the band structure, which enable both direct and indirect transitions. The dual-band gap structure highlights the synergistic interaction between V_2_O_5_ and ZrO_2_, enhancing the photocatalytic efficiency of the material under various light conditions.

Similarly, the Bi_2_S_3_-4%@ZrO_2_@cellulose composite exhibited a direct band gap of 3.9 eV and an indirect band gap of 1.5 eV, both of which are lower than those of the ZrO_2_@cellulose and V_2_O_5_-modified composites. Bi_2_S_3_, a narrow bandgap material, is likely responsible for this reduction, as it introduces states within the band gap that facilitate lower energy electronic transitions. The reduced indirect band gap, in particular, suggests that Bi_2_S_3_ creates favorable pathways for charge carriers, improving the composite’s photocatalytic properties.

In contrast, the ZnO-4%@ZrO_2_@cellulose composite displayed a direct band gap of 4.5 eV and an indirect band gap of 2.82 eV. ZnO, known for its wide bandgap, did not significantly alter the direct band gap when compared to the other composites but did cause a reduction in the indirect band gap. The presence of ZnO likely maintains the overall high energy requirement for direct electronic transitions but contributes to the indirect transition through its electronic structure. This suggests that ZnO primarily influences the electronic properties by altering the overall charge dynamics within the composite.

The addition of PANI to the ZrO_2_@cellulose composite resulted in a direct band gap of 4.17 eV and an indirect band gap of 1.6 eV. PANI, a conducting polymer, typically narrows the band gap of semiconducting composites due to its semiconducting properties. The reduction in the indirect band gap here indicates that PANI interacts with the ZrO_2_@cellulose matrix, likely facilitating charge transfer and promoting lower energy electronic transitions.

Lastly, the MoS_2_-4%@ZrO_2_@cellulose composite showed a direct band gap of 4.2 eV and an indirect band gap of 3.46 eV. MoS_2_, a material known for its direct band gap properties, helps to reduce the indirect band gap of the composite. The relatively smaller difference between the direct and indirect gaps in MoS_2_-modified composites suggests enhanced electronic interactions between MoS_2_ and the ZrO_2_@cellulose matrix.

### Photocatalytic activity

The performance of ZrO_2_ @ Cellulose and different fixed bed photocatalysts (PCs), including ZnO-4%@ZrO_2_@Cellulose, V_2_O_5_-4%@ZrO_2_@Cellulose, Bi_2_S_3_-4%@ZrO_2_@Cellulose, MoS_2_-4%@ZrO_2_@Cellulose, and PANI-4%@ZrO_2_@Cellulose, was investigated for the photoreduction of Cr (IV) metal from simulated wastewater under simulated sunlight irradiation.

The adsorptive behavior of Cr (VI) was first studied by conducting reactions under dark conditions. It was observed that ZrO_2_@cellulose and V_2_O_5_@ZrO_2_@cellulose exhibited poor adsorption behavior for Cr (VI), with no decrease in the level perceived after 60 min of contact time. MoS_2_@ZrO_2_@cellulose showed only 18% removal of Cr (VI) upon adsorption. However, ZnO@ZrO_2_@cellulose and Bi_2_S_3_@ZrO_2_@cellulose fibers demonstrated remarkable 30% and 40% adsorption rates, respectively. This indicates a higher affinity for Cr (VI) adsorption, which can significantly influence subsequent photocatalytic activity.

Additionally, control experiments conducted under a solar simulator without fixed bed PCs showed no change in the level of Cr (IV) metal after 60 min of light irradiation. This suggests that the spontaneous Cr (VI) photolysis under simulated solar irradiation is negligible.

**Fig. 7:**
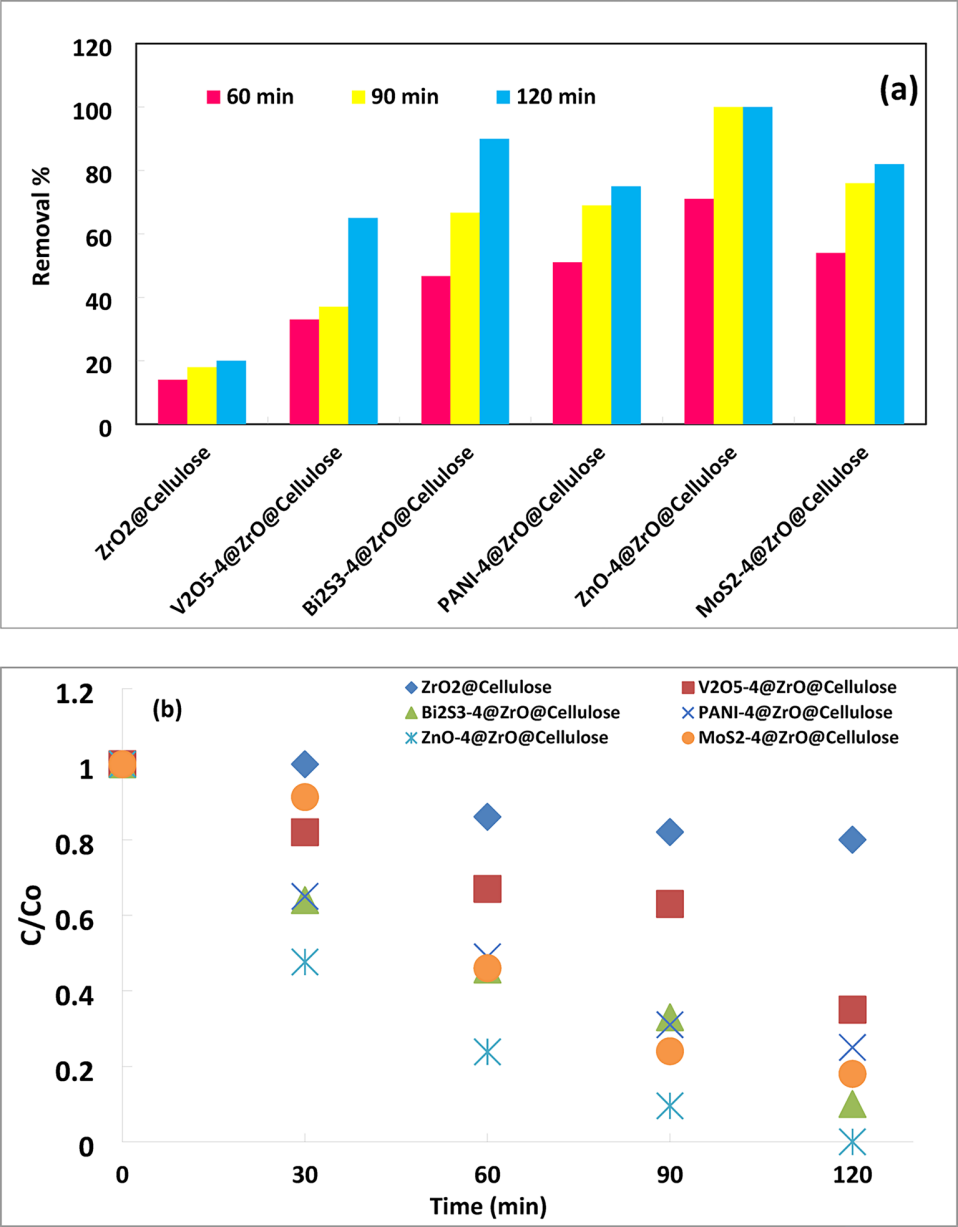

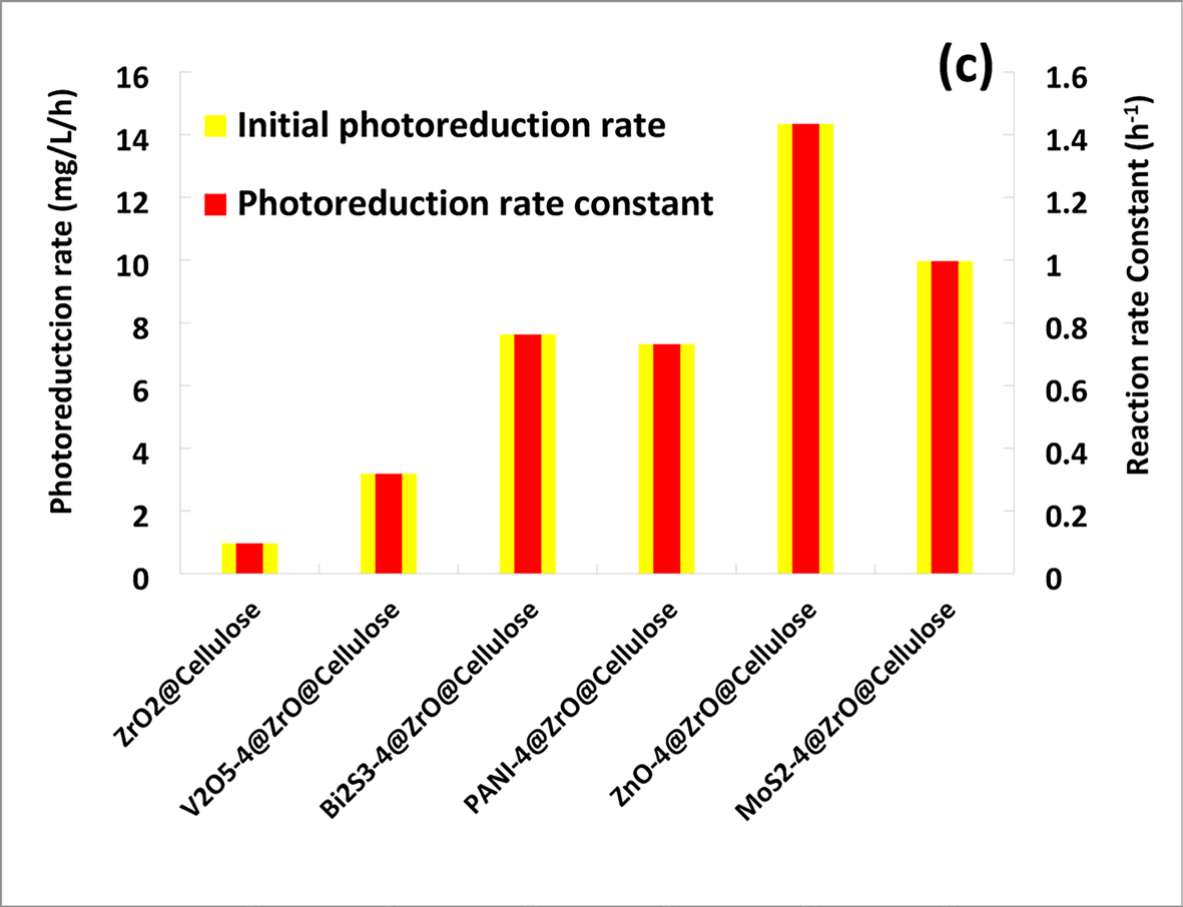
(**a**) Photocatalytic removal of (Cr (VI) Conc. of 10 ppm) over different photocatalytic materials supported over ZrO_2_@Cellulose under simulated solar irradiation light (PC dose = 1000 ppm), (**b**) the kinetics for the photocatalytic reduction process over different fixed bed PC fibers under simulated solar light, (**c**) photoreduction rate constant and the reaction rate constant over different fixed bed PC fibers under simulated solar light.

Experimental results of Cr (VI) ions photoreduction using different fixed beds under simulated sunlight irradiation are illustrated in Fig. 7a. Remarkably, ZnO-4%@ZrO_2_@cellulose fibers and Bi_2_S_3_-4%@ZrO_2_@cellulose fibers achieved approximately 100% and 90% photoreduction of Cr (VI) ions, respectively, after 120 min of irradiation. MoS_2_-4%@ZrO_2_@cellulose fibers showed an intermediate photocatalytic reduction, achieving 82% reduction after 120 min of irradiation In contrast, PANI-4%@ZrO_2_@cellulose fibers and V_2_O_5_-4%@ZrO_2_@cellulose fibers exhibited lower reduction rates of only 75% and 65%, respectively, during the same period. This finding is very high compared with the activity of ZrO_2_@cellulose which achieved only 20% after the same photoreduction time.

The enhanced photocatalytic efficiency of ZrO_2_@cellulose through the incorporation of ZnO, V_2_O_5_, Bi_2_S_3_, MoS_2_, and PANI Photocatalysts can be attributed to their superior photocatalytic properties, which are further amplified by synergistic interactions with the ZrO_2_@cellulose substrate. These photocatalysts extend the material’s light absorption range, promote effective charge carrier separation, and facilitate the generation of reactive oxygen species (ROS). Collectively, these effects contribute to the enhanced reduction of Cr (VI) ions, improving the overall photocatalytic performance.

The highest reduction activity observed for ZnO-4%@ZrO_2_@cellulose fibers and Bi_2_S_3_-4%@ZrO_2_@cellulose fibers suggests that fibers with higher adsorption capacity tend to exhibit superior photocatalytic performance. In addition, this can be attributed to their lower band gaps, enhanced light absorption, and increased surface area, all of which collectively contribute to improved photocatalytic efficiency.

**Fig. 8:**
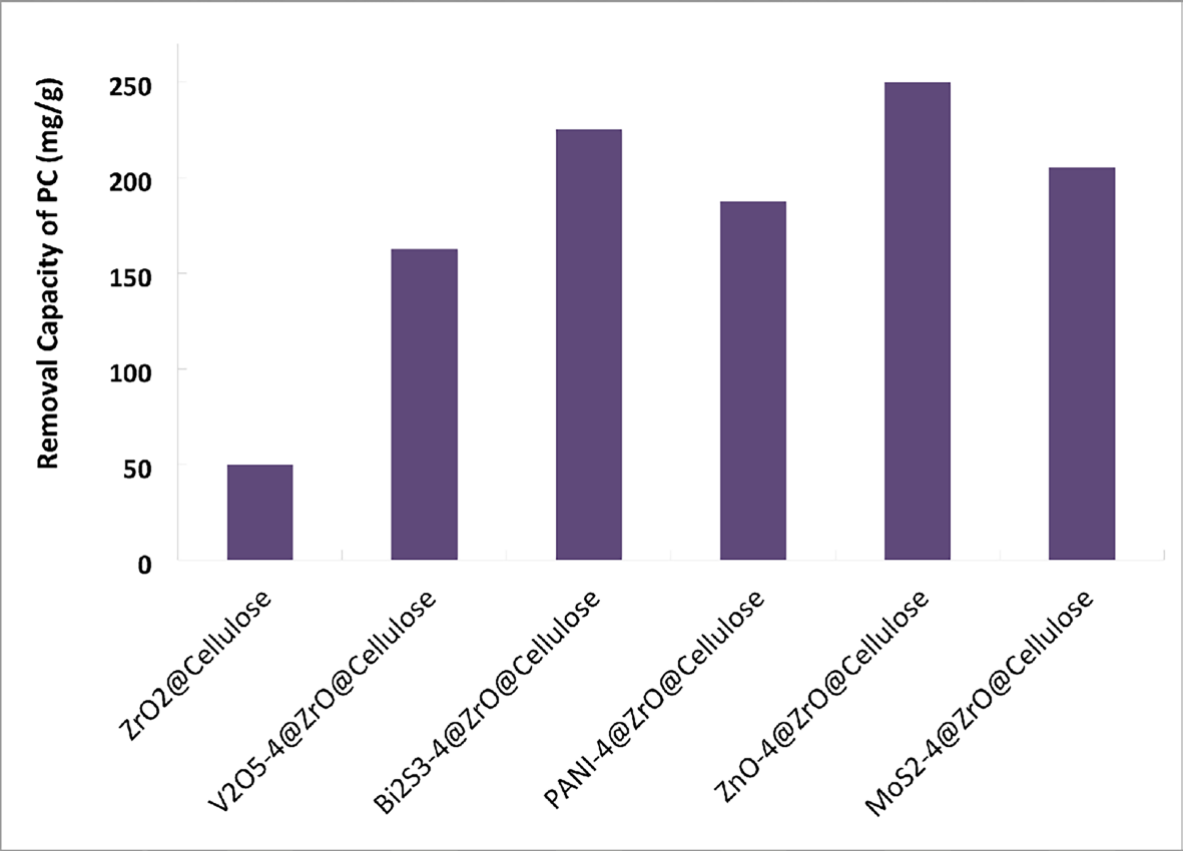
Normalized photocatalytic reduction capacity of Cr ^6+^ metal ions using different fixed bed PCs support on cellulose fibers under simulated sunlight.

Figure 7b shows the kinetics of photocatalytic reduction of Cr (VI) over different fixed bed PC fibers under simulated solar light. The results obtained confirm that photocatalytic reduction of Cr (VI) obeys the first-order kinetic reaction. ZnO-4%@ZrO_2_@cellulose fibers PC has a photoreduction rate for Cr (VI) higher 15 times than that of control ZrO_2_@cellulose fibers PC. Meanwhile, Bi_2_S_3_-4%@ZrO_2_@cellulose fibers, MoS_3_-4%@ZrO_2_@cellulose fibers, PANI-4%@ZrO_2_@cellulose, and V_2_O_5_-4%@ZrO_2_@cellulose fibers have photoreduction rates for Cr (VI) higher 8, 10, 7.6, and 2.6 times that of ZrO_2_@cellulose fibers PC, respectively. In addition, the photoreduction rate constant and the reaction rate constant over different fixed bed PC fibers have been illustrated in Fig. 7c. Moreover, to estimate the quantum yield for each fixed bed PC, the photocatalytic capacity was calculated for actual PC mass (i.e. 4%). Figure 8 shows the normalized capacity of photocatalytic reduction for Cr (VI). It was found that ZnO-4%@ZrO_2_@cellulose, Bi_2_S_3_-4%@ZrO_2_@cellulose, and MoS_2_-4%@ZrO_2_@cellulose fibers PC have the higher removal capacity, which is 5, 4.5, and 4 folds higher than that of control PC (Fig. 8). The higher activity for ZnO-4%@ZrO_2_@cellulose fibers and Bi_2_S_3_-4%@ZrO_2_@cellulose is attributed to high surface area and high adsorptive activity. Subsequently, the evident enhancement of photocatalytic activity for ZnO-4%@ZrO_2_@cellulose fibers and Bi_2_S_3_-4%@ZrO_2_@cellulose help inhibit electron-hole pair recombination and enhance interfacial charge transfer reactions. In addition, Bi_2_S_3_, and ZnO fixed beds PC showed higher surface adsorption for Cr (VI) ions that increases the available ions which also increases the photocatalytic activity as well as increases reduction^53^. Meanwhile, the moderate photoreduction removal rate of Cr (VI) for PANI-4%@ZrO_2_@cellulose and V_2_O_5_-4%@ZrO_2_@cellulose fibers is owing to lower adsorption activity for metal despite high surface area (See Table 1).

**Fig. 9:**
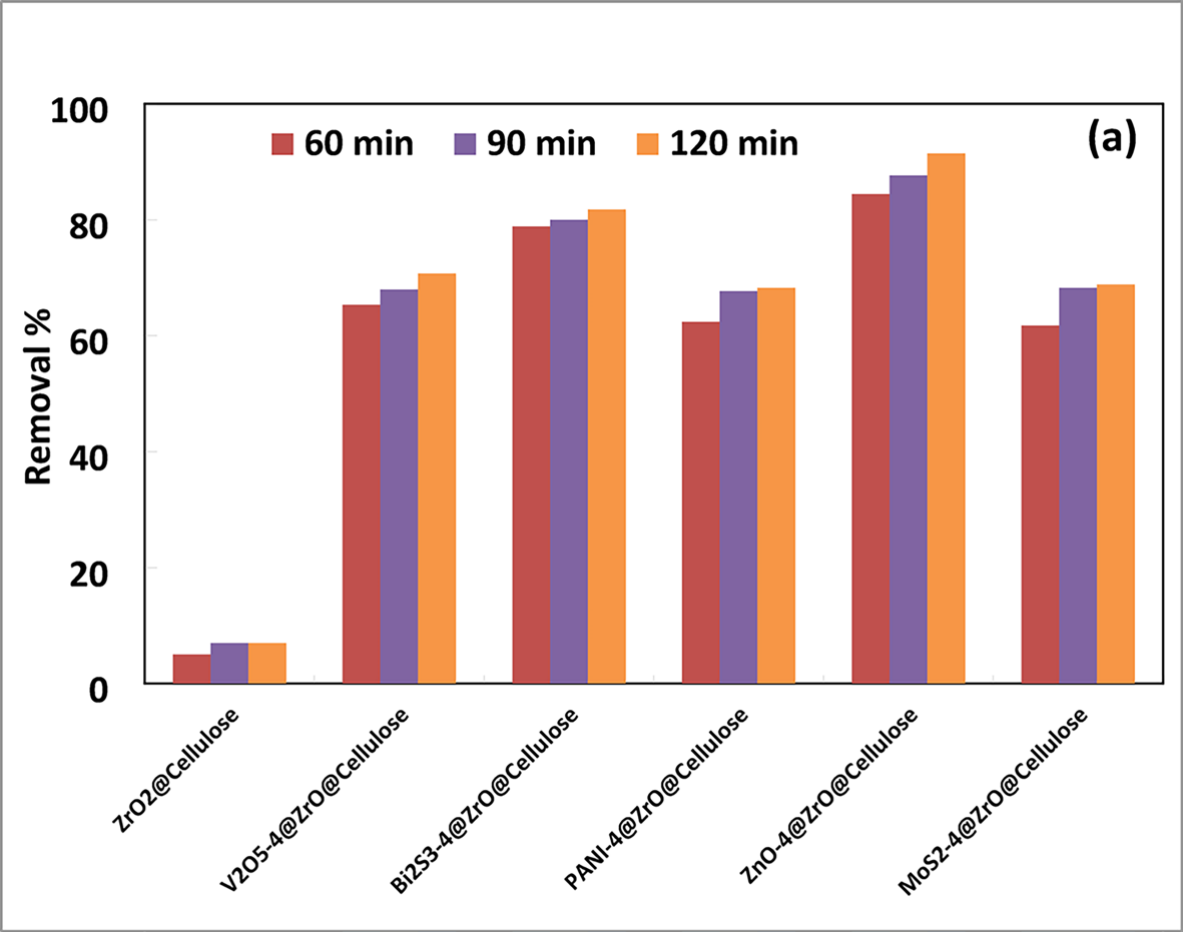

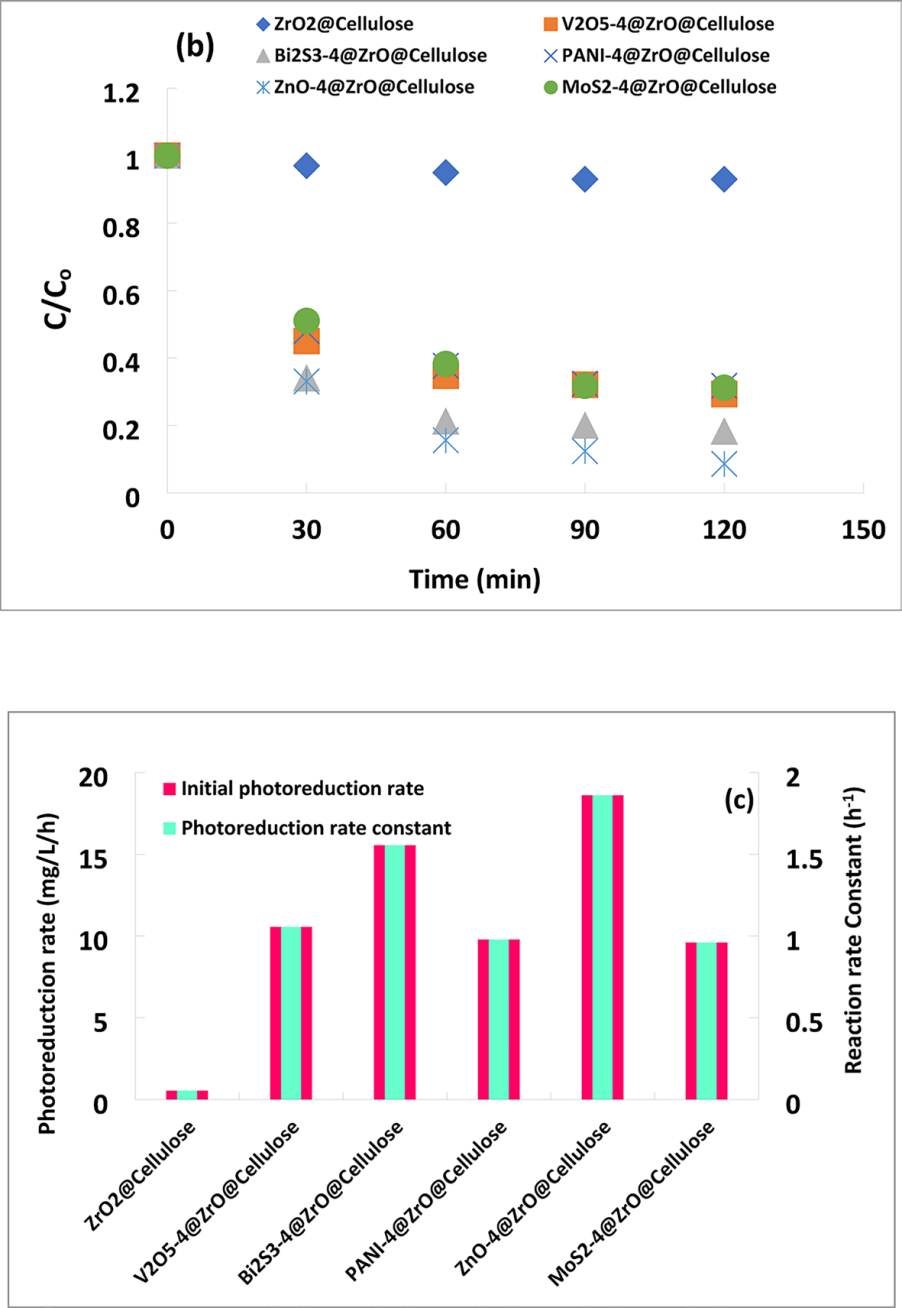
(**a**) Photocatalytic removal of (Cu (II) Conc. of 10 ppm) over different photocatalytic materials supported over ZrO_2_@Cellulose under simulated solar irradiation light (PC dose = 1000 ppm), (**b**) the kinetics for the photocatalytic reduction process over different fixed bed PC fibers under simulated solar light, (**c**) photoreduction rate constant and the reaction rate constant over different fixed bed PC fibers under simulated solar light.

In addition, the different fixed bed photocatalysts supported on cellulose fibers were also tested for the photoreduction of copper ions Cu(III) from simulated wastewater at natural pH. The initial concentration of copper ions was 25 ppm, with a catalyst dose of 1000 ppm under solar simulator irradiation. As depicted in Fig. 9, the photoreduction of Cu(III) exhibited varying efficiencies across the different fixed bed composites. Notably, the ZnO-4%@ZrO_2_@cellulose fibers and Bi_2_S_3_-4%@ZrO_2_@cellulose fibers photocatalysts demonstrated high photoreduction rates, achieving removal values of 91% and 82%, respectively. In comparison, the V_2_O_5_-4%@ZrO_2_@cellulose, MoS_2_-4%@ZrO_2_@cellulose, and PANI-4%@ZrO_2_@cellulose fibers exhibited lower removal rates of 71%, 69%, and 68%, respectively, after 120 min of irradiation time. These results underscore the superior performance of Bi_2_S_3_ and ZnO-based nanocomposites in reducing the concentration of Cu (III) ions in wastewater.

**Fig. 10:**
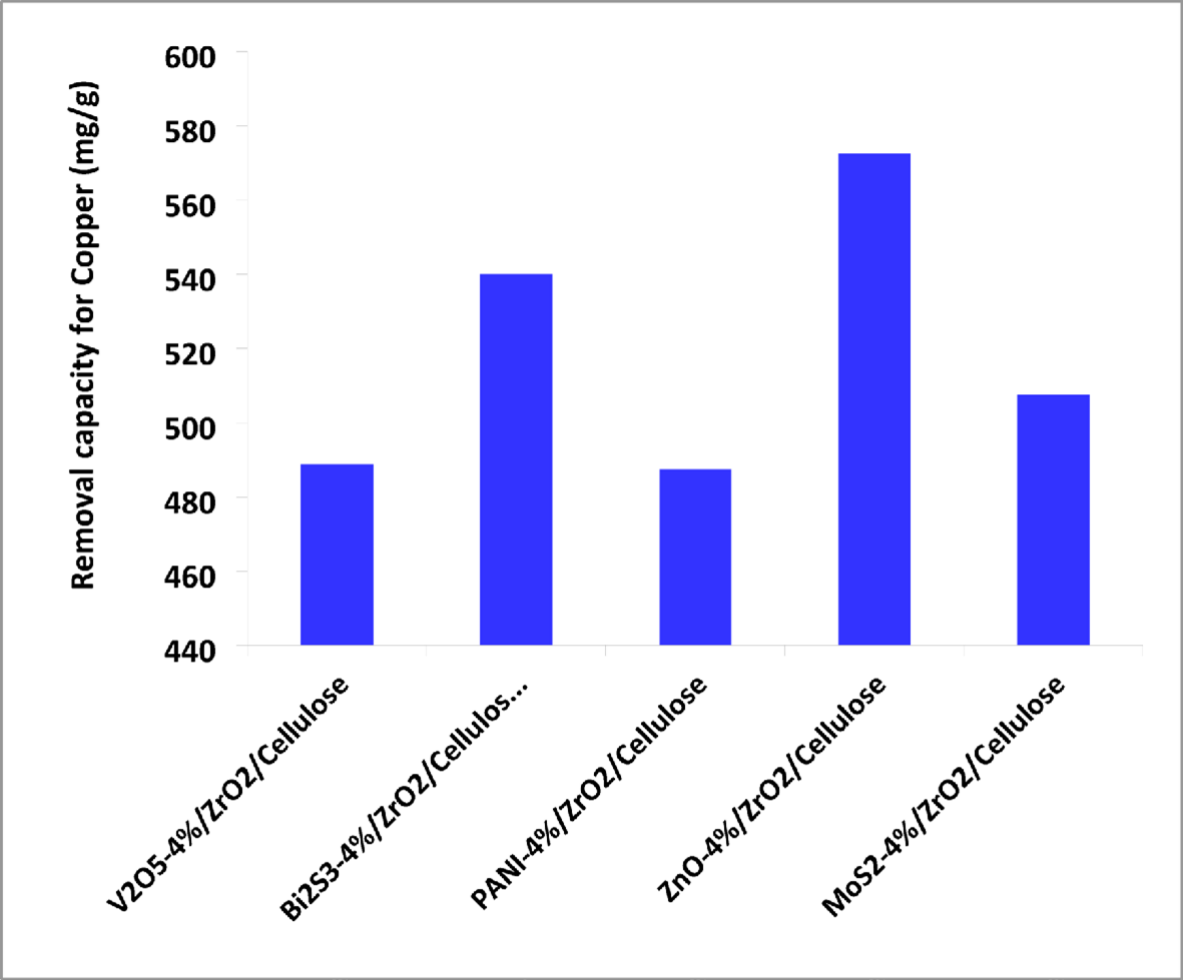
Normalized photocatalytic reduction capacity of Cu (III) metal ions using different fixed bed PCs support on cellulose fibers under simulated sunlight.

Furthermore, the first-order fitted kinetic curves depicted in Fig. 9b illustrate the photocatalytic reduction of Cu (III) ions across various fixed-bed PCs. The photoreduction rate constants for Cu (III) ions ranged from 0.054 to 1.86 h^− 1^. Notably, the ZnO-4%@ZrO_2_@cellulose and Bi_2_S_3_-4%@ZrO_2_@cellulose fibers photocatalysts exhibited photoreduction rate constants 34 and 28.8 times higher than that of ZrO_2_@cellulose fibers, respectively. Additionally, the recorded photoreduction rate constants were 20, 18, and 18 times higher than those of the control for V_2_O_5_-4%@ZrO_2_@celluloses, MoS_2_-4%@ZrO_2_@cellulose, and PANI-4%@ZrO_2_@cellulose fibers, respectively. This heightened activity is attributed to the enhanced surface area or improved optical properties of the fixed bed PCs. Bi_2_S_3_ and ZnO-based composites may exhibit stronger surface adsorption affinity towards copper ions compared to other materials like V_2_O_5_, MoS_2_, and PANI. This enhanced adsorption capacity allows more copper ions to come into contact with the photocatalyst surface, thereby increasing the efficiency of the photoreduction process. Also, Bi_2_S_3_ and ZnO nanoparticles possess optimal bandgap energies for efficient absorption of solar irradiation, enabling them to generate electron-hole pairs effectively. This characteristic ensures a continuous supply of reactive species for the reduction reaction. Figure 10 shows the photocatalytic normalized capacity rate of reduction for copper ions using the photocatalytic process. It was found that ZnO-4%@ZrO_2_@cellulose fibers photocatalyst has a higher removal capacity of 528 mg/g/h which is tenfold higher than that of the ZrO_2_-4%@cellulose.

In contrast, the lower removal rates observed for V_2_O_5_-4%@ZrO_2_@cellulose, MoS_2_-4%@ZrO_2_@cellulose, and PANI-4%@ZrO_2_@cellulose fibers may be attributed to their comparatively lower photocatalytic efficiency, limited light absorption capacity, or weaker surface interactions with copper ions.

### Photodegradation mechanism

**Fig. 11:**
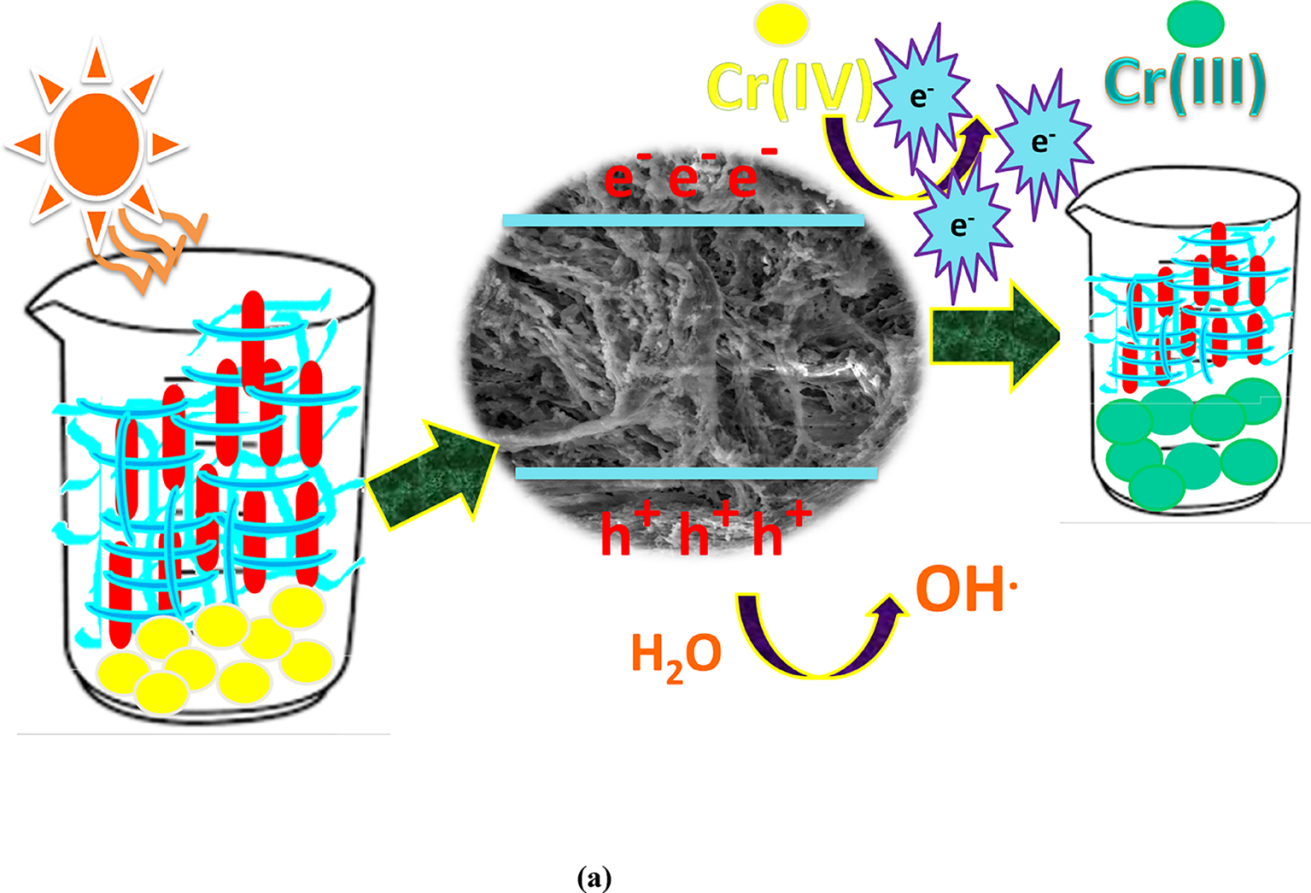

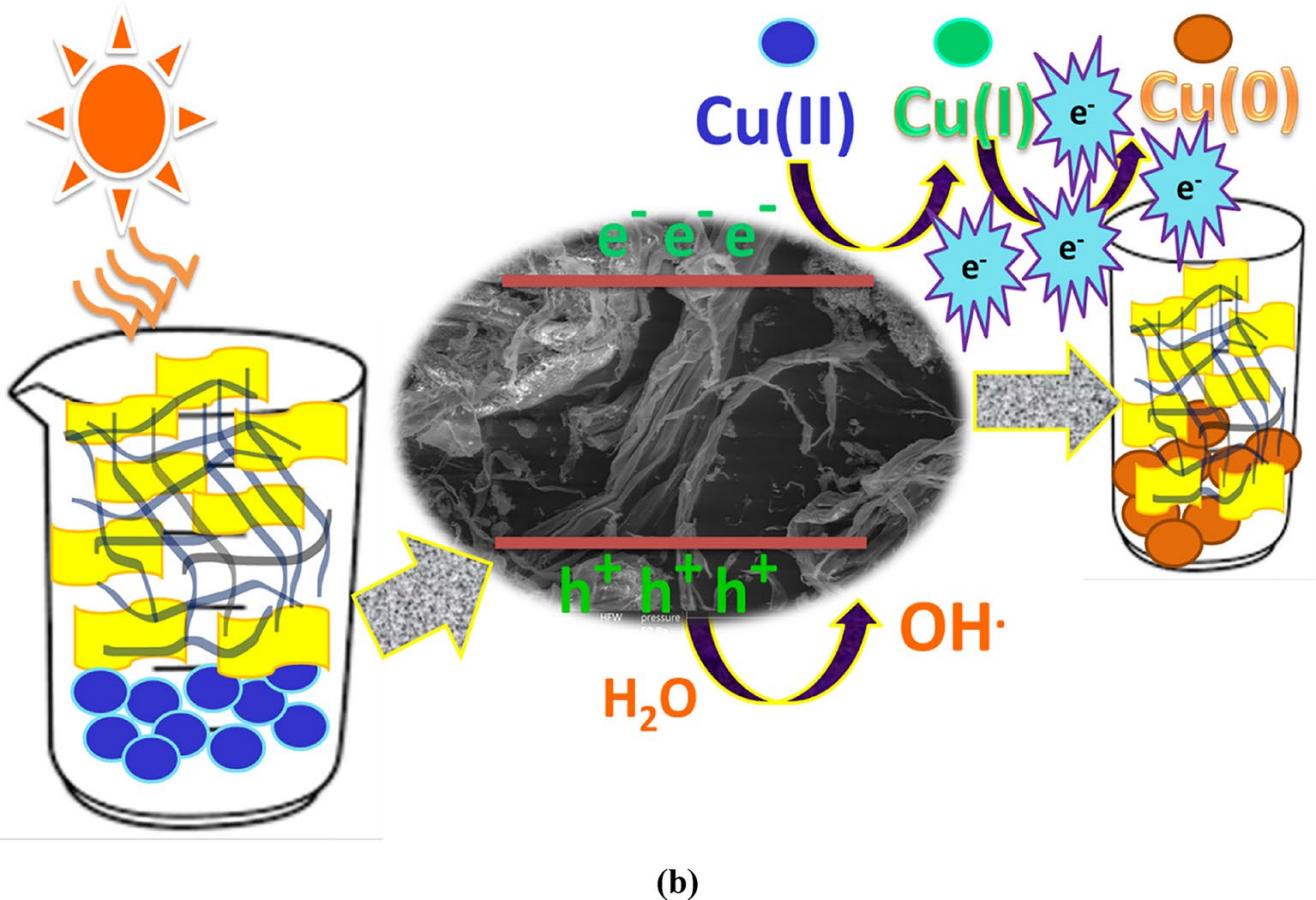
The simulated mechanism for the reduction of Cr (IV) and Cu (II) utilizing ZnO-4%@ ZrO_2_@cellulose and Bi_2_S_3_-4%@ZrO_2_@cellulose fiber, respectively.

Figure 11a, b illustrates schematic diagrams of the photoreduction processes for Cr(VI) and Cu(III) ions, respectively, using ZnO-4%@ZrO₂@cellulose and Bi₂S₃-4%@ZrO₂@cellulose fiber photocatalysts under simulated sunlight. These diagrams exemplify the prepared fixed bead fibers. Simulated solar irradiation acts as the energy source, activating the ZnO and Bi₂S₃ nanoparticles supported on ZrO₂ nanoparticles, which are immobilized on cellulose fibers.

Upon absorbing sunlight, the ZnO and Bi₂S₃ nanoparticles generate electron-hole pairs, with the photogenerated electrons (e⁻) and holes (h⁺) driving subsequent redox reactions. The excited electrons reduce Cr (VI) and Cu (III) ions to less toxic forms—Cr (III) and Cu (0), respectively—via electron transfer. Meanwhile, reactive oxygen species (ROS), such as superoxide radicals (•O₂⁻), are produced as intermediates, enhancing the reduction process. These mechanisms highlight the efficiency and potential of the fixed bead fiber photocatalysts in detoxifying heavy metal ions.

### Photocatalytic microbial removal

The photocatalytic removal of different microbial species, including E. coli, Salmonella spp, and Bacillus spp, was investigated using the various fixed bed fibers, namely ZrO_2_@cellulose, ZnO-4%@ZrO_2_@cellulose, V_2_O_5_-4%@ZrO_2_@cellulose, Bi_2_S_3_-4%@ZrO_2_@cellulose, MoS_2_-4%@ZrO_2_@cellulose, and PANI-4%@ZrO_2_@cellulose, under solar simulator irradiation with a dose of 1 mg/mL for 2 h. In dark experiments, all prepared samples showed low activity for the deactivation of E. coli, salmonella spp, and, Bacillus spp.

**Fig. 12:**
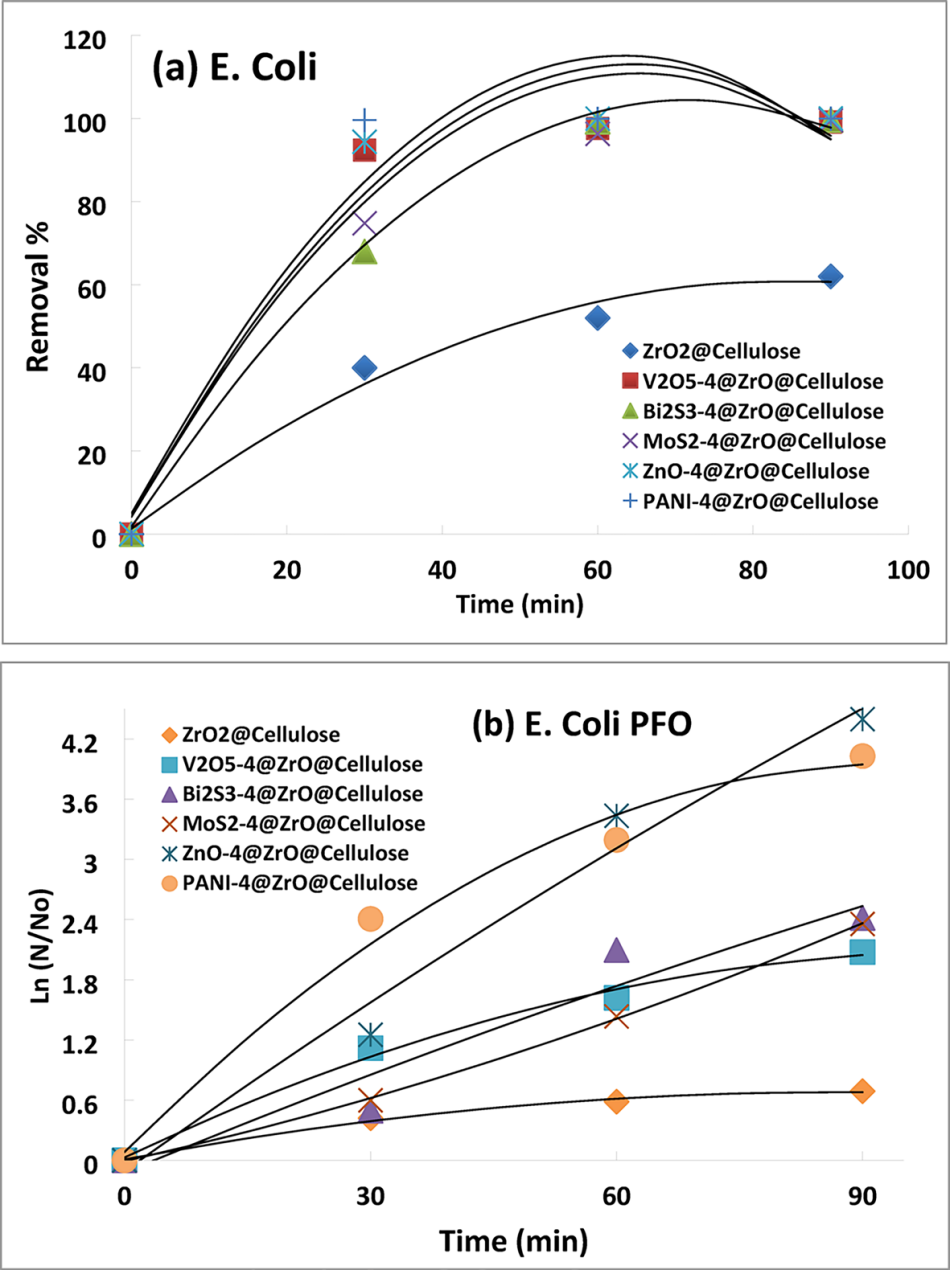
Photocatalytic inactivation of E. Coli (**a**) microbial removal rate, (**b**) PSFO kinetic model.

**Fig. 13:**
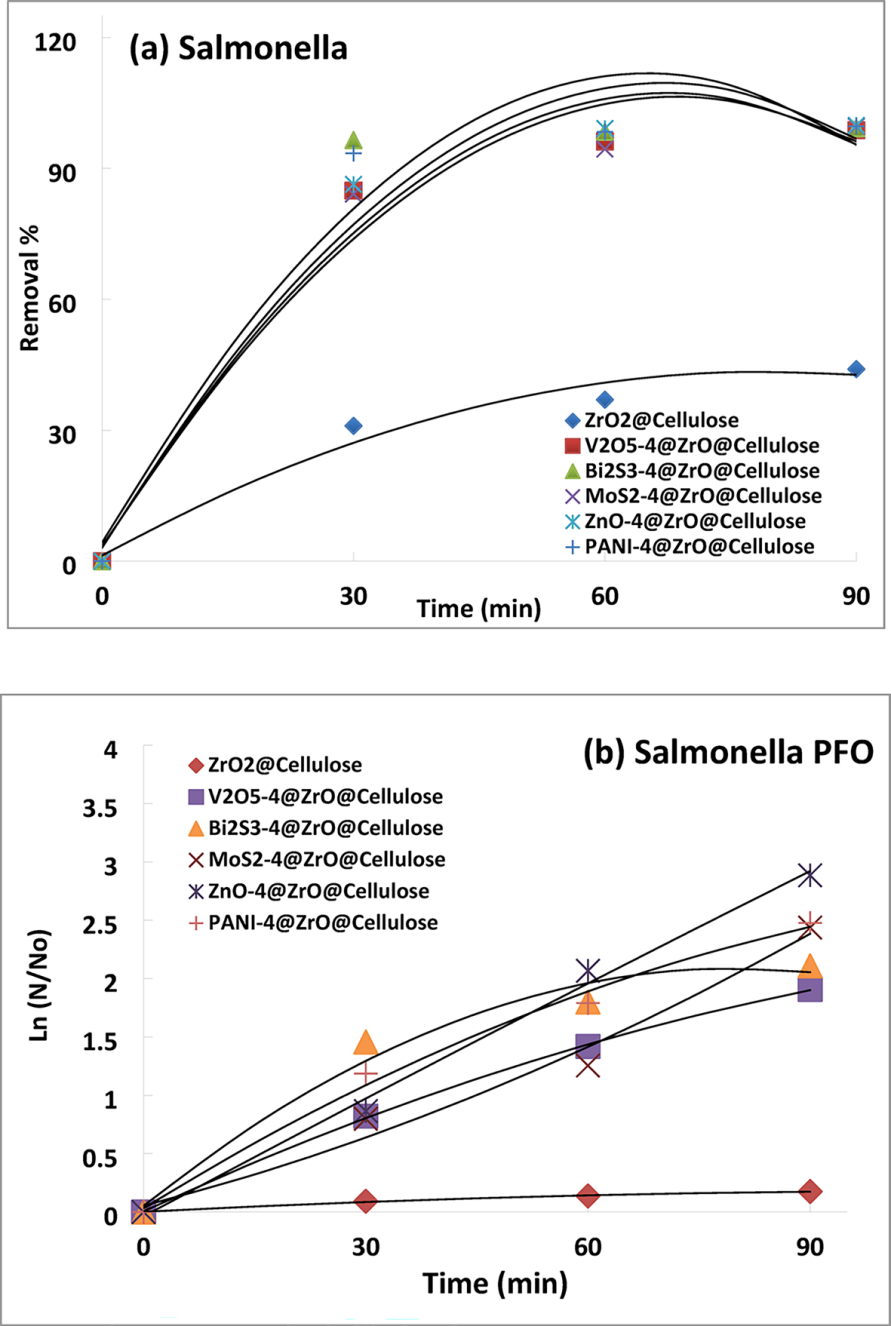
Photocatalytic inactivation of Salmonella (**a**) microbial removal rate, (**b**) PSFO kinetic.

**Fig. 14:**
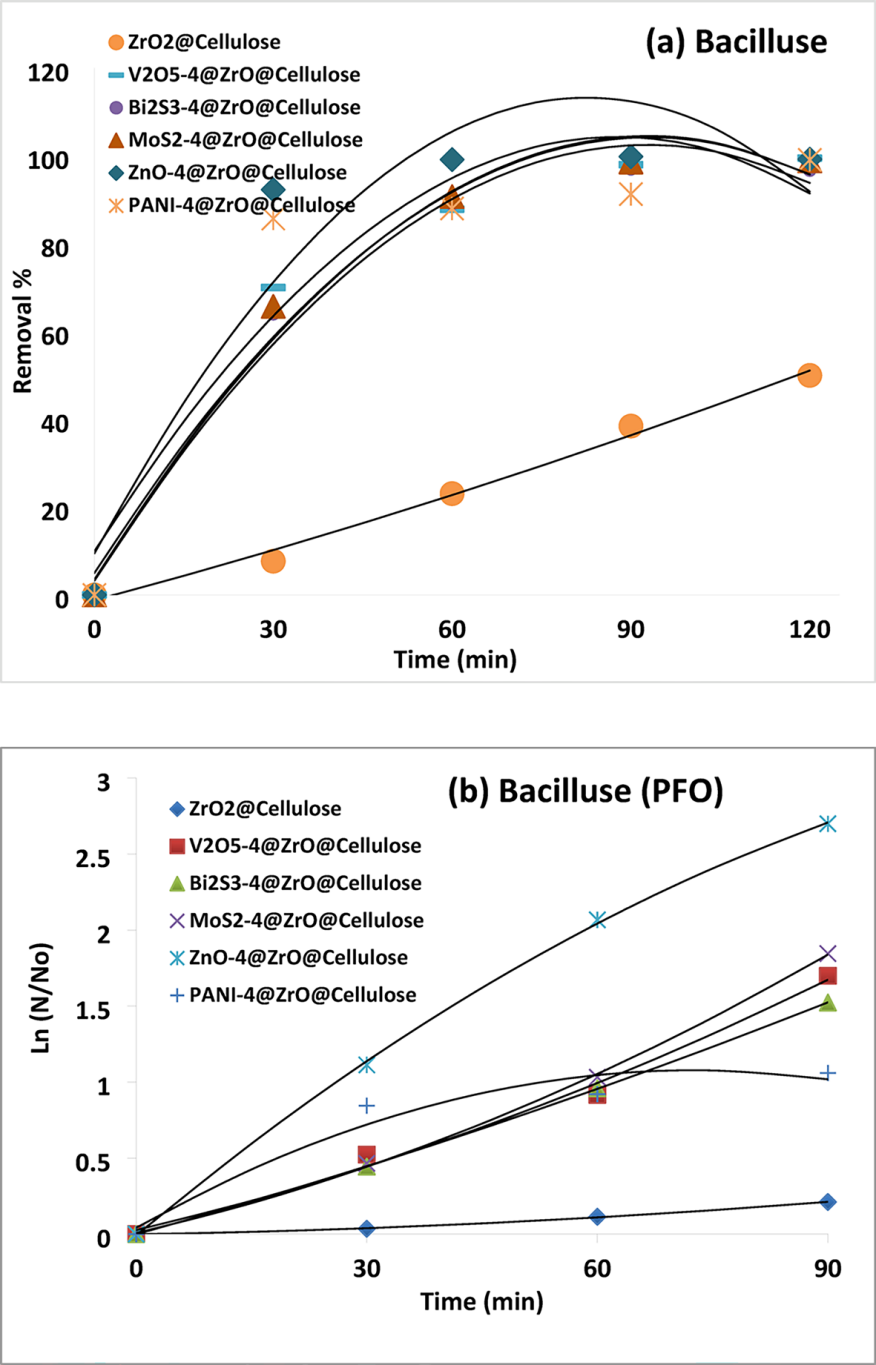
Photocatalytic inactivation of Bacillus (**a**) microbial removal rate, (**b**) PSFO kinetic.

However, under simulated solar irradiation, significant differences in photocatalytic microbial removal efficiency were observed among the different fixed bed fibers as depicted in Figs. 12 and 13, and 14.

For E. coli, ZrO_2_@cellulose exhibited a moderate removal rate, with recorded removal rates of 40%, 52%, and 62% after 30, 60, and 90 min, respectively. In contrast, the other fixed beds, including ZnO-4%@ZrO_2_@cellulose, V_2_O_5_-4%@ZrO_2_@cellulose, Bi_2_S_3_-4%@ZrO_2_@cellulose, MoS_2_-4%@ZrO_2_@cellulose, and PANI-4%@ZrO_2_@cellulose, demonstrated higher deactivation rates compared to ZrO_2_@cellulose. Specifically, ZnO-4%@ZrO_2_@cellulose, V_2_O_5_-4%@ZrO_2_@cellulose, Bi_2_S_3_-4%@ZrO_2_@cellulose, MoS_2_-4%@ZrO_2_@cellulose, and PANI-4%@ZrO_2_@cellulose achieved deactivation rates of 100%, 99%, 99%, 99.6%, and 100%, respectively, after 90 min of solar simulator irradiation (Fig. 12a). Also, Fig. 12b; Table 2 illustrate the PFO kinetic rates for the different fixed beads of cellulosic fiber in the photocatalytic removal of E. coli.

The degradation kinetics of E. coli was investigated using various fixed-bead fibers, aiming to evaluate their efficacy in microbial degradation. The degradation kinetics were found to conform to the pseudo-first-order (PFO) kinetic model, exhibiting high correlation coefficients (R^2^ ≈ 0.99) (Table 2), indicative of the reliability of the model for describing the degradation process.

Notably, the degradation rates of PANI-4%@ZrO_2_@Cellulose, ZnO-4%@ZrO_2_@Cellulose, V_2_O_5_-4%@ZrO_2_@Cellulose, Bi_2_S_3_-4%@ZrO_2_@Cellulose, and MoS_2_-4%@ZrO_2_@Cellulose were determined to be 5.8, 3.8, 2.6, 2.2, and 1.2 times higher than that of ZrO_2_@Cellulose, respectively. Table 3 summarizes selected recent studies on photocatalysts used for metal removal, providing a comparative perspective on their performance and applicability.

The results indicate that the fixed bead fibers incorporating catalytic nanomaterials, such as ZnO NRs, V_2_O_5_ and PANI NPs, Bi_2_S_3,_ and MoS_2_ NSHs, showed superior efficacy in degrading E. coli bacteria compared to pristine ZrO_2_@Cellulose. This enhanced degradation performance can be attributed to the synergistic effects of these composite materials. Among the tested fibers, PANI-4%@ZrO_2_@Cellulose demonstrated the highest degradation rate, underscoring its potential as a potent antimicrobial agent.

Similarly, for Salmonella spp, ZnO-4%@ZrO_2_@cellulose, V_2_O_5_-4%@ZrO_2_@cellulose, Bi_2_S_3_-4%@ZrO_2_@cellulose, MoS_2_-4%@ZrO_2_@cellulose, and PANI-4%@ZrO_2_@cellulose exhibited high photocatalytic removal efficiencies (Fig. 13a), with removal rates of 99.9%, 99.6%, 99%, 99.6%, and 99.9%, respectively, after 90 min. Notably, ZrO_2_@cellulose showed lower photocatalytic deactivation for Salmonella spp, with a removal rate of 44% after the same time.

Figure 13b and Table 2 investigated the first-order kinetic degradation rates of various fixed bead fibers in degrading Salmonella bacteria, comparing them to ZrO_2_@Cellulose. Results showed significant enhancements in degradation rates for Bi_2_S_3_-4%@ZrO_2_@Cellulose, PANI-4%@ZrO_2_@Cellulose, ZnO-4%@ZrO_2_@Cellulose, V_2_O_5_-4%@ZrO_2_@Cellulose, and MoS_2_-4%@ZrO_2_@Cellulose, with rates 15.6, 12.5, 10.6, 9.5 times higher than ZrO_2_@Cellulose.

Furthermore, the photocatalytic activity for the removal of Bacillus spp was investigated (Fig. 14). The results showed microbial deactivation rates of 38%, 100%, 97%, 99%, 98%, and 99% over ZrO_2_@cellulose, MoS_2_-4%@ZrO_2_@cellulose, Bi_2_S_3_-4%@ZrO_2_@cellulose, ZnO-4%@ZrO_2_@cellulose, PANI-4%@ZrO_2_@cellulose and V_2_O_5_-4%@ZrO_2_@cellulose, respectively.

**Fig. 15:**
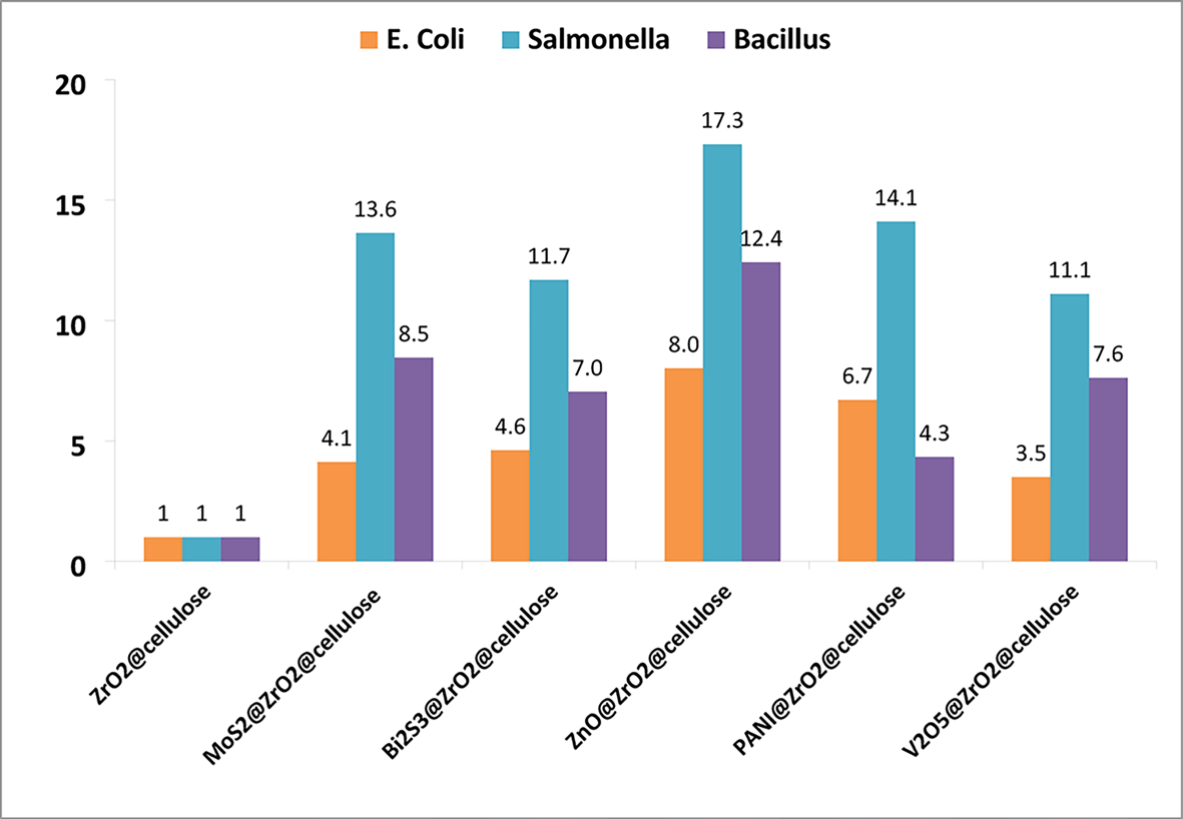
Relative normalized photocatalytic inactivation rate constants over PC-fixed bed materials for different bacterial species.

In addition, notably, the ZnO-4%@ZrO_2_@cellulose composite demonstrated the highest normalized photocatalytic inactivation rate constants as shown in Fig. 15.

Overall, these findings underscore the potential of the investigated fixed bed fibers as effective photocatalysts for the removal of various microbial contaminants from water under visible light irradiation.

**Table 2:** The photocatalytic degradation kinetics (PFO kinetics) of the various prepared fixed bed photocatalysts (PCs) against three different types of microbial.

	K app (min^-1^)		
	E. Coli	Salmonella	Bacillus
ZrO_2_@cellulose	0.0153	0.0032	0.0008
MoS_2_-4%@ZrO_2_@cellulose	0.018	0.016	0.0116
Bi_2_S_3_-4%@ZrO_2_@cellulose,	0.034	0.05	0.014
ZnO-4%@ZrO_2_@cellulose,	0.058	0.034	0.042
PANI-4%@ZrO_2_@cellulose,	0.0822	0.05	0.0285
V_2_O_5_-4%@ZrO_2_@cellulose	0.04	0.029	0.01183

The disinfection process under simulated solar irradiation observed in this study can be attributed to the photocatalytic activity of the fixed bed fiber and the generation of reactive oxygen species (ROS), such as hydroxyl radicals (^•^OH) and superoxide radicals (^•^O_2_^−^) upon irradiation, which possess strong oxidative properties capable of damaging the cellular structures of microbial contaminants.

**Fig. 16:**
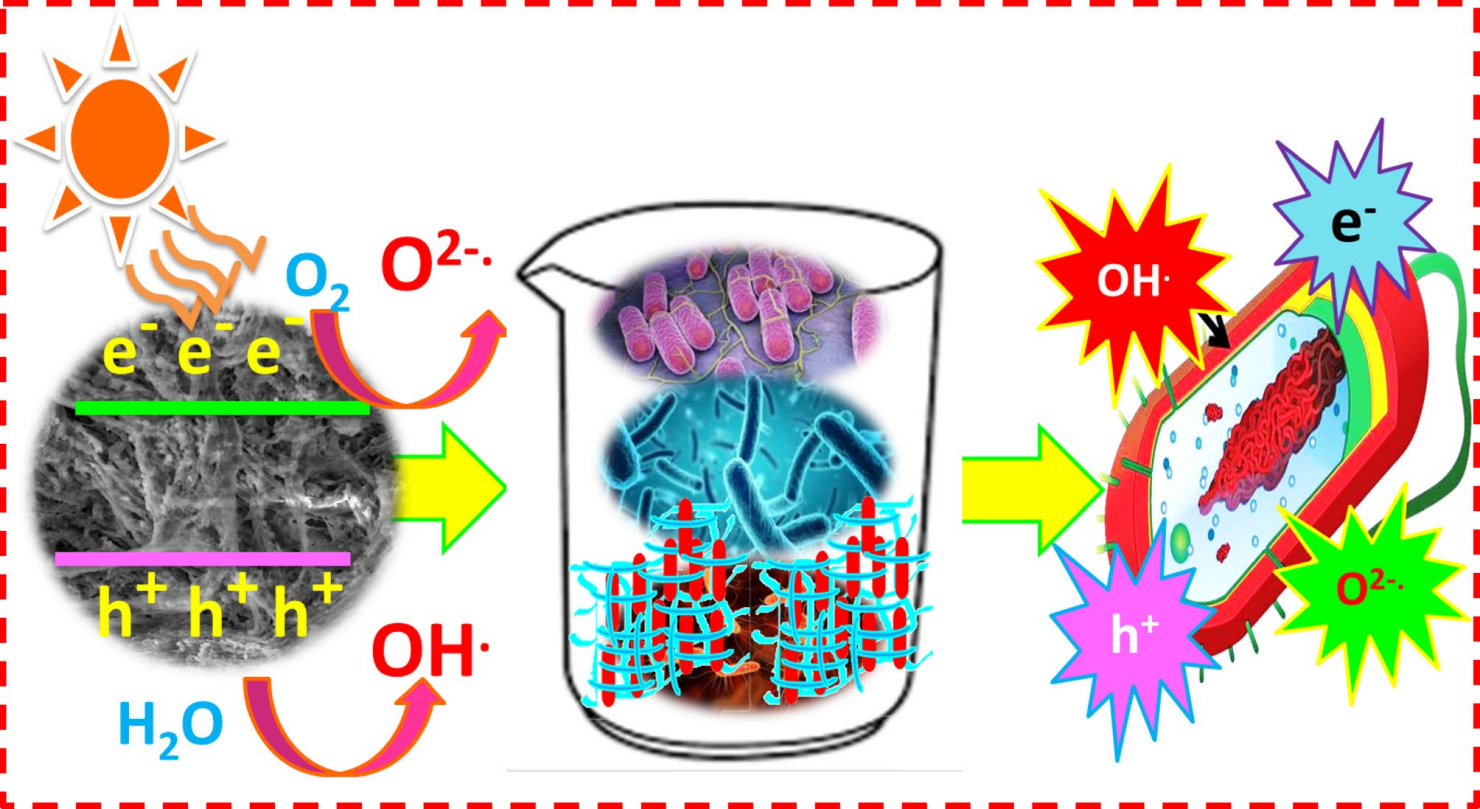
Schematic diagram of the proposed mechanism for photocatalytic removal of different bacterial species.

Several proposed mechanisms elucidate the photocatalytic removal of bacteria as simulated in Fig. 16. One pathway involves reactive oxygen species (ROS) attacking the bacterial cell membrane, penetrating the cell to oxidize macromolecular substances like proteins, which ultimately leads to cell death. Alternatively, ROS can induce oxidation and damage to coenzyme A upon attacking the cell membrane, causing it to transform into a dimeric state. This alteration disrupts respiration processes reliant on coenzyme A, resulting in cell death. Additionally, ROS can oxidize cell membranes, causing their breakdown alongside cell walls, leading to the release of intracellular macromolecules, such as proteins, and cations like potassium (K^+^), ultimately leading to bacterial cell death^54^.

Furthermore, the differences in the removal efficiencies observed for different microbial species, such as E. coli, Salmonella spp, and Bacillus spp, can be attributed to cell structure variations and oxidative damage susceptibility. Overall, the results demonstrate the effectiveness of the tested fixed bed fibers as photocatalytic agents for the disinfection of water contaminated with various microbial species under simulated solar irradiation.

As shown in Table 2, the developed ZnO-4%@ZrO₂@cellulose and Bi₂S₃-4%@ZrO₂@cellulose photocatalysts achieved excellent disinfection performance under simulated sunlight, with E. coli inactivation exceeding 97%. These results are comparable to or even surpass those reported for other advanced materials such as AgNPs–g-C₃N₄/TiO₂ and Cu₂O@MoS₂–TiO₂. The high microbial reduction is attributed to the effective generation of reactive oxygen species (ROS) and the synergistic interaction between ZrO₂, cellulose fibers, and the loaded photocatalysts. These findings emphasize the strong potential of the developed fixed-bed systems for practical, efficient, and sustainable water disinfection applications.

To highlight the effectiveness of the developed photocatalyst, Table 4 provides a comparison with other photocatalysts reported in the literature for microbial disinfection applications.

**Table 3:** Comparison of heavy metal removal using various photocatalysts reported in the literature.

Photocatalyst	Target Ion(s)	Light Source	Removal Efficiency (%)	Time (min)	Reference
ZnO-4%@ZrO₂@Cellulose	Cr(VI), Cu(II)	Simulated sunlight	94.2 (Cr), 91.7 (Cu)	60	This work
Bi₂S₃-4%@ZrO₂@Cellulose	Cr(VI), Cu(II)	Simulated sunlight	92.8 (Cr), 89.5 (Cu)	60	This work
ZnO–g-C₃N₄ composite	Cr(VI)	Visible light	91.3	120	Chemosphere, 2023, 342, 140,181
Biochar@TiO₂	Cr(VI)	UV light	88.5	90	Journal of Cleaner Production, 2023, 398, 136,549
Fe₃O₄@MoS₂/GO	Cu(II)	Sunlight	87.6	120	Chemical Engineering Journal, 2022, 446, 137,273

**Table 4:** Comparison of photocatalysts for microbial disinfection reported in literature.

Photocatalyst	Target Microorganism(s)	Light Source	Disinfection Efficiency (%)	Time (min)	Reference
ZnO-4%@ZrO₂@Cellulose	E. coli	Simulated sunlight	99.3	60	This work
Bi₂S₃-4%@ZrO₂@Cellulose	E. coli	Simulated sunlight	97.8	60	This work
Black TiO₂₋ₓ	S. aureus	Visible light	100	420–480	^55^
Fe₂O₃–AgBr	E. coli, S. aureus	Visible light (LED)	> 99	Not specified	^56^
C₇₀–TiO₂ Hybrid	E. coli O157:H7	Visible light (≥ 420 nm)	~ 100	120	PMC Article: PMC4861983
Ag/AgBr/LDH	E. coli	Visible light (≥ 400 nm)	100	80	PubMed ID: 36,623,654
Cu–TiO₂ Nanocomposite	E. coli, S. aureus	Visible light (405 nm LED)	≥ 99.9	Not specified	ACS Omega
Fe–Cd/TiO₂	E. coli	Visible light	99.9	45	Mehrzad Feilizadeh et al., Applied Catalysis B: Environmental, 2015, 168–169, 441–447. DOI:10.1016/j.apcatb.2014.12.034
Ag@ZnO Core–Shell	E. coli, S. aureus	Sunlight	100	60–90	MDPI Article
Ag–AgCl/ZnFe₂O₄	E. coli	Visible light	Not specified	Not specified	Upreti et al., Environmental Science and Pollution Research, 2018, 25, 9331–9341. DOI:10.1007/s11356-018-1225-x
PThC/TiO₂	E. coli, MRSA	White LED light	100	30–180	MDPI Catalysts

## Conclusion

In conclusion, the study has successfully demonstrated the fabrication and characterization of fixed bed photocatalysts comprising ZrO_2_@cellulose composite matrices loaded with various photocatalytic materials. Through comprehensive characterization techniques such as XRD analysis, FTIR spectroscopy, SEM imaging, N_2_ adsorption/desorption isotherms, and diffuse reflectance spectroscopy, the structural, chemical, and optical properties of the composite materials were elucidated. These analyses confirmed the effective integration and distribution of photocatalytic materials within the cellulose matrix, highlighting the photocatalysts’ strong interactions and efficient immobilization.

Furthermore, the photocatalytic performance of the developed fixed bed photocatalysts was evaluated for the remediation of metal ions, specifically Cr(VI) and Cu(II), in simulated wastewater under simulated sunlight irradiation. The results revealed exceptional photocatalytic activity, with ZnO-4%@ZrO_2_@cellulose and Bi_2_S_3_-4%@ZrO_2_@cellulose fibers exhibiting high removal efficiencies for both Cr (VI) and Cu (II) ions. Notably, the superior performances were attributed to the high surface area, strong adsorption capacity, and optimal bandgap energies of ZnO and Bi_2_S_3_ nanoparticles, facilitating the efficient generation of reactive species and charge transfer processes. This study underlines the potential of fixed bed photocatalysts in microbial deactivation under simulated solar irradiation. Enhanced removal efficiencies observed for E. coli, Salmonella spp, and Bacillus spp highlight the efficacy of composite materials, particularly ZnO-4%@ZrO_2_@cellulose, V_2_O_5_-4%@ZrO_2_@cellulose, Bi_2_S_3_-4%@ZrO_2_@cellulose, MoS_2_-4%@ZrO_2_@cellulose, and PANI-4%@ZrO_2_@cellulose. The accurate description of microbial degradation rates using the pseudo-first-order kinetics model further confirms the efficacy of fixed bed fibers.

Overall, the developed fixed-bed photocatalysts offer promising prospects for the efficient purification of water resources contaminated with heavy metals and organic pollutants. Their green, cost-effective, and scalable nature makes them suitable for addressing water scarcity and sanitation challenges, particularly in resource-limited settings.

## Data Availability

“The datasets used and/or analysed during the current study available from the corresponding author on reasonable request”.
